# Unraveling *p*-Cresol: from biosynthesis to biological and biochemical activities

**DOI:** 10.3389/fphar.2025.1665421

**Published:** 2025-09-22

**Authors:** Ying Zhang, Xinfang Xu, Xiaona Fan, Yuting Wu, Xiufang Zhang, Yingxue Wu, Xiangri Li, Qinwen Zou

**Affiliations:** ^1^ Beijing Lianxin Pharmaceutical Co., Ltd., Beijing, China; ^2^ School of Chinese Materia Medica, Beijing University of Chinese Medicine, Beijing, China

**Keywords:** *p*-cresol, biosynthesis, biological activity, biochemical activity, toxicity

## Abstract

*p*-Cresol, a ubiquitous low-molecular-weight volatile phenolic compound, permeates the vicinity of human, plant, and animal ecosystems through complex biosynthetic pathways and environmental exposure routes. Beyond its well-documented toxicity and metabolic interference, emerging evidence reveals its paradoxical roles as a pivotal signaling molecule in diverse ecological and biological systems. In the context of emerging research on the intricate interplay between chemical substances and life processes, its multifaceted biological and biochemical activities have become a focal point of interdisciplinary exploration. This comprehensive review systematically dissects the latest advancements in *p*-cresol research, meticulously tracing its biosynthesis pathways. Beyond its well-documented pungent odor and toxicity thresholds, the review delves into the cutting-edge discoveries of *p*-cresol’s potential as an antioxidant and modulator of glucose homeostasis. Notably, the discussion on *p*-cresol removal strategies includes modulating the microbiota, optimizing diet and improving activated charcoal adsorption. By bridging the gaps between basic research and practical applications, this review not only provides a holistic understanding of *p*-cresol’s dual-edged nature but also offers strategic guidance for developing safer and more efficient *p*-cresol utilization models while mitigating its health risks, ultimately guiding precision probiotic interventions for gut microbiota modulation (to lower endogenous *p*-cresol production) and targeted clinical trials for metabolic disorder management (e.g., chronic kidney disease-related *p*-cresol accumulation).

## 1 Introduction

**FIGURE 1:**
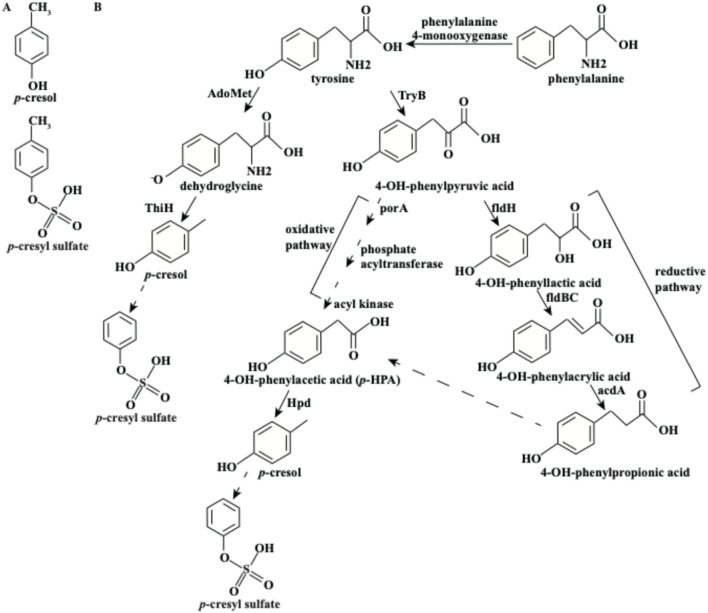
Structure of *p*-cresol and *p*-cresyl sulfate **(A)** and its biosynthetic pathways **(B)**.

*p*-Cresol (4-methylphenol) belongs to the category of volatile organic methyl phenols (Al Hinai et al., 2019). Chemically, *p*-cresol is a monohydroxybenzene derivative with a methyl group at the para position of the phenol ring (Figure 1A), which underpins its physical properties (e.g., volatility) and biological activities. With a molecular weight of 108.1 Da, *p*-cresol exhibits high permeability and can penetrate both the intestinal barrier and blood-brain barrier (Stachulski et al., 2023). Environmental exposure is one source of *p*-cresol, including rainwater, petroleum, solvents, perfumes, and cosmetics (Persico and Napolioni, 2013). In humans, another significant source of *p*-cresol exposure is the decomposition of tyrosine by intestinal anaerobic bacteria, particularly *Clostridium difficile* (Harrison et al., 2022). The metabolic pathway of tyrosine to *p*-cresol has been extensively studied in recent years.

Characterized by pungent “horsy”, “burnt leather”, “animal stench” and “sweaty saddle”-like off-odors (Du et al., 2017), *p*-cresol is a notorious taint in foods, such as Chinese liquor, cheese, bacon, smoked foods and beverages (Du et al., 2017; Ji et al., 2020). Paradoxically, under controlled conditions and appropriate amounts, it can be used as a food flavoring in the Chinese Standard for the Use of Food Additives (GB2760-2024) (National Food Safety Standard: Standard for the Use of Food Additives, 2024) and the Research Institute for Fragrance Materials database (http://fragrancematerialsafetyresource.elsevier.com) (Api et al., 2021), reflecting its complex risk-benefit profile.

Toxicologically, *p*-cresol exhibits dose-dependent hazards: excessive exposure triggers headaches, rashes, itching, anemia, and various neurological disorders (Jia-ying and Jin, 2022), with an oral lethal dose (LD_50_) of 20–5,000 mg/kg/day (Brial et al., 2020). Its classification as a possible human carcinogen by the United States Environmental Protection Agency (EPA) (National Center for Environmental Assessment, 1988) and WHO’s 1 μg/L potable water limit (Singh et al., 2008) highlight regulatory concerns. Despite this, emerging evidence reveals pharmacological potential: antioxidant activity against low-density lipoprotein oxidation (Ujhelyi et al., 2006a; 2006b), selective antimicrobial effects (Gram-negative inhibition) (Passmore et al., 2018), and metabolic benefits in type 2 diabetes (glucose homeostasis, β-cell stimulation at non-toxic doses) (Brial et al., 2020). Ecologically, *p*-cresol serves as an estrus-specific signal in livestock species (buffaloes (Manikkaraja et al., 2022) or mares (Karthikeyan et al., 2014)) and a communication cue in carnivores (lions (Soso and Koziel, 2017)), underscoring its evolutionary significance.

Given this dichotomy of toxicity and utility, this review systematically examines *p*-cresol’s biosynthesis pathways, biological and biochemical mechanisms, and clearance strategies. By integrating these domains, we aim to inform safer regulatory thresholds and innovative applications, bridging knowledge gaps in toxicology, pharmacology, and environmental science.

## 2 Review methodology

### 2.1 Databases and search strategy

Literature was retrieved from PubMed, Web of Science, Scopus, and Google scholar using keywords (“cresol”, “*p*-cresol”, “para-cresol”, “4-cresol”, “*ρ*-cresol”, “4-methylphenol”, “*p*-methyl phenol”) and Boolean operators (“AND/OR”) for articles published between 2001 and 2025. We prioritized studies from 2020–2025 to ensure timeliness.

### 2.2 Inclusion/exclusion criteria

Included studies were peer-reviewed original research, reviews, or meta-analyses focused on *p*-cresol’s biosynthesis, biological activities, or clinical relevance; excluded studies were conference abstracts, non-English articles, or those with irrelevant focus.

### 2.3 Data extraction and synthesis

Key data (mechanisms, study models, main findings) were extracted by two independent authors, with discrepancies resolved via discussion.

## 3 The biosynthesis of *p*-cresol

There are two biosynthetic pathways for *p*-cresol by microorganisms, including *Escherichia coli, Clostridium sporogenes, C. difficile* or other intestinal bacteria (Figure 1B; Dodd et al., 2017; Harrison et al., 2021; Kriek et al., 2007; Saito et al., 2018). The first pathway involves the direct cleavage of tyrosine by ThiH (tyrosine lyase) (Saito et al., 2018). Specifically, tyrosine is first metabolized into dehydroglycine by Radical *S*-adenosylmethionine (AdoMet). ThiH then cleaves the Cα-Cβ bond of dehydroglycine to produce *p*-cresol (Kriek et al., 2007). Notably, this pathway is the initial step in the biosynthesis of thiamine, with *p*-cresol being formed as a by-product (Saito et al., 2018).

The second pathway for *p*-cresol synthesis is more complex and involves the decomposition of tyrosine through the intermediate 4-OH-phenylacetic acid (*p*-HPA). Initially, tyrosine is metabolized into 4-OH-phenylpyruvic acid by tyrosine aminotransferase (TyrB). This compound can then be converted into *p*-HPA via either an oxidative or reductive pathway (Norman et al., 2022).

• In the oxidative pathway, 4-OH-phenylpyruvic acid is oxidatively decarboxylated by pyruvate: ferredoxin oxidoreductase A (porA). Subsequent reactions involving phosphate acyltransferase and acyl kinase lead to the formation of *p*-HPA.
• In the reductive pathway, 4-OH-phenylpyruvic acid is reduced to 4-OH-phenyllactic acid by phenyllactate dehydrogenase (fldH). This compound is then dehydrated to 4-OH-phenylacrylic acid by phenyllactate dehydratase (fldBC). Finally, 4-OH-phenylacrylic acid is reduced to 4-OH-phenylpropionic acid by acyl-CoA dehydrogenase (acdA) and subsequently converted to *p*-HPA (Dodd et al., 2017).

In both pathways, *p*-HPA is ultimately decarboxylated into *p*-cresol by HpdBCA decarboxylase (Harrison et al., 2021). It is worth noting that certain microorganisms can also convert exogenous *p*-HPA into *p*-cresol; for instance, under antibiotic exposure, *C. difficile* upregulates enzymes involved in *p*-HPA decarboxylation, thereby enhancing *p*-cresol production to inhibit competing gut commensals and support its colonization in the colon (Harrison et al., 2021; Harrison et al., 2022). Additionally, phenylalanine can be hydroxylated into tyrosine by phenylalanine 4-monooxygenase, which can then lead to *p*-cresol synthesis through the pathways described above (Gryp et al., 2017).

## 4 The biological activities of *p*-cresol

**FIGURE 2:**
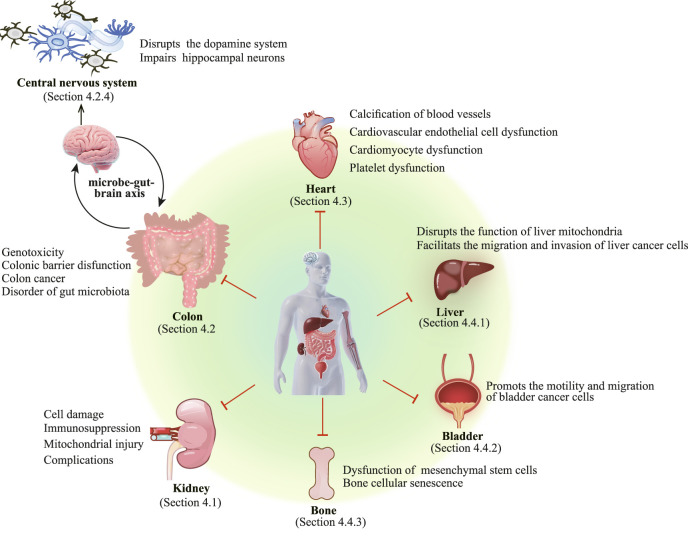
*p*-Cresol exhibits toxic effects on a variety of human organs and tissues, including the kidneys, colon, liver, heart, bladder, and bones. Additionally, it can cause damage to the central nervous system via the microbe-gut-brain axis.

Gut bacteria play a crucial role in maintaining human health (Gryp et al., 2017), and are particularly responsible for the assimilation of amino acids (Di Paola et al., 2023). The colon microbiota transforms tyrosine and phenylalanine into protein-bound uremic retention solutes, such as *p*-cresol. Subsequently, these solutes are excreted by the glomerulus in the form of urine (Blachier and Andriamihaja, 2022). When renal elimination function is impaired, the composition of the intestinal microbiome undergoes alterations, leading to a disruption in metabolic homeostasis (Mlynarska et al., 2024). As a result, *p*-cresol accumulates largely, especially in patients with chronic kidney disease (CKD) (Blachier and Andriamihaja, 2022). Existing studies have found *p*-cresol can be detected in blood and feces (Blachier and Andriamihaja, 2022). Known as a uremic toxin, *p*-cresol exhibits significant biological activities and exerts detrimental effects on multiple human organs or tissues, such as kidney, colon, heart, central nervous cells, liver, bladder, and bone (Figure 2).

### 4.1 Impacts of *p*-cresol on the kidney

**FIGURE 3:**
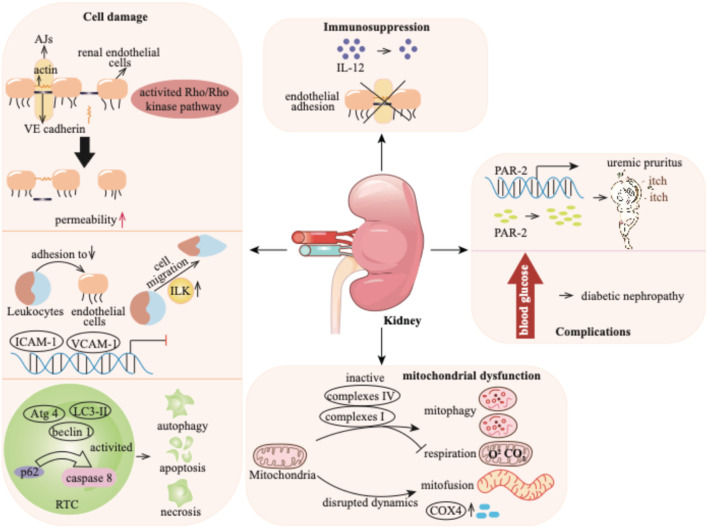
*p*-Cresol exhibits detrimental effects on the kidney by causing cell damage, compromising the immune system and mitochondrial function, and finally fosters downstream complications ranging from uremic pruritus to the progression of diabetic nephropathy. AJs: adherens junctions; VE: vascular endothelial; RTC: renal tubular cell; ICAM-1: intercellular adhesion molecule-1; VCAM-1: vascular cell adhesion molecule-1; PAR-2: protease-activated receptor 2; COX4: cytochrome c oxidase subunit 4.

The glomerular filtrate contains numerous water-soluble uremic toxins, such as *p*-cresol. However, *p*-cresol is primarily protein-bound, with 90% of it binding to plasma proteins. This binding limits its filtration by the glomerulus, as large protein-bound molecules typically do not pass through the glomerular filtration barrier (Poesen et al., 2016). Thus, only free *p*-cresol is filtered by the glomerulus. The bound fraction of *p*-cresol is mainly secreted by tubular epithelial cells. Both free and bound fractions of *p*-cresol are ultimately excreted in the urine (Gryp et al., 2017). The kidney is the primary target organ of *p*-cresol in CKD patients. The translocation of *p*-cresol into the systemic circulation can lead to a variety of detrimental effects on the kidney, such as cell damage, immunosuppression, mitochondrial injury, or potentially contribute to the development of complications (Figure 3).

#### 4.1.1 Cell damage

*p*-Cresol has effects on various cell types, including renal endothelial cells, monocytes, and renal tubular cells (RTCs). The endothelial cell barrier plays a critical role in regulating the exchange of plasma proteins and circulating cells through endothelial cell-to-cell junctions (Lugano et al., 2023). This barrier function is mediated by the small GTPase Rho protein and Rho kinase, which maintain actin cytoskeleton organization and thereby preserve endothelial barrier integrity (Krogt et al., 2023). *p*-Cresol significantly increases the endothelial permeability through activating the Rho/Rho kinase pathway. This effect is demonstrated by the fact that the specific Rho kinase inhibitor Y-27632 markedly attenuates *p*-cresol-induced increases in endothelial permeability (Cerini et al., 2004). The endothelial cell barrier is also involved in adherens junctions (AJs), which are constituted by vascular endothelial (VE)-cadherin, a membrane-bound protein. VE-cadherin is connected to the actin cytoskeleton (Nan et al., 2023), and its proper localization is essential for endothelial barrier function. *p*-Cresol disrupts the actin cytoskeleton and alters AJs by reducing the dense peripheral band of junctional F-actin, inducing stress fiber formation, and relocalizing VE-cadherin away from cell-to-cell junctions. These changes result in the formation of visible gaps between adjacent endothelial cells, thereby increasing endothelial permeability (Cerini et al., 2004).

Leukocytes have been utilized to investigate the cellular dysfunction caused by uremia (Vanholder et al., 2008). When an infection occurs, leukocytes adhere to endothelium, cross the endothelial barrier, and trigger an immune response (Arts et al., 2021). The process is mediated by endothelial adhesion molecules (EAMs), such as intercellular adhesion molecule-1 (ICAM-1), vascular cell adhesion molecule-1 (VCAM-1) and E-selectin. Inflammatory cytokines, including tumor necrosis factor (TNF) or interleukin-1*ß* (IL-1*ß*), can induce the production of adhesion molecules (Kihara et al., 2021; Versele et al., 2022). *p*-Cresol, on its own, has no direct impact on the expression of EAMs. However, it has been found to inhibit cytokine-induced stimulation and the mRNA expression of ICAM-1 and VCAM-1, but not E-selectin. TNF or IL-1*ß* stimulates the adhesion of monocyte (e.g., THP-1) to endothelial cells, Conversely, *p*-cresol can reduce this THP-1 adhesion. Additionally, *p*-cresol can induce monocyte migration. Integrin-linked kinase (ILK), a kinase involved in cell migration, is activated at the protein level in THP-1 cells by *p*-cresol. Subsequently, ILK participates in the transmigration of THP-1 cells by mediating the remodeling of F-actin and podosome formation (Campillo et al., 2022).

Evidence has demonstrated the cytotoxic effects of *p*-cresol on RTCs in a concentration-dependent manner in patients with CKD (Brocca et al., 2013). *p*-Cresol elicits a toxic reaction and induces cell death through pathways of apoptosis and necrosis, especially at high concentrations. The apoptosis of RTCs has been morphologically observed during the progression of CKD (Maremonti et al., 2022). The loss of cell membrane permeability caused by *p*-cresol is likely the primary cause of necrosis (Brocca et al., 2013). In addition to apoptosis and necrosis, autophagy, which can be induced by toxins, also contributes to cell death (Lin et al., 2015). It has been reported that *p*-cresol can reduce the proliferation of renal proximal tubular cells by significantly increasing cell apoptosis, which is accompanied by the activation of autophagy. The signaling adaptor p62 serves as a signaling hub that determines cell survival and apoptosis, and its interaction with caspase 8 can trigger apoptotic cell death (Lee et al., 2021). *p*-Cresol leads to the accumulation of p62, followed by the activation of caspase 8-induced cell apoptosis (Lin et al., 2015). Moreover, treatment with *p*-cresol upregulates autophagy markers such as LC3-II, beclin 1, and Atg 4 (Lin et al., 2015).

#### 4.1.2 Immunosuppression

Interleukins (ILs), predominantly secreted by macrophages, exhibit regulatory activity on immunocompetent cells (Mansurov et al., 2022). In patients with CKD, immune dysregulation is often a concomitant condition, leading to elevated levels of ILs (Glorieux et al., 2020). Existing evidence has indicated that *p*-cresol can inhibit the production of IL-12 in a dose-dependent manner (Kawakami et al., 2009). Moreover, *p*-cresol has been demonstrated to suppress cytokine-induced endothelial adhesion (Flynn et al., 2025). IL-12 plays a crucial role in immune responses (Wang et al., 2025), and endothelial adhesion is an essential step in the inflammatory process (Varinder et al., 2023). By inhibiting the production of IL-12 and cytokine-induced endothelial adhesion, *p*-cresol disrupts normal immune signaling and the recruitment of immune cells. These findings suggest that *p*-cresol contributes to the immunodeficiency observed in CKD patients by impeding the responses of endothelial cells to inflammatory cytokines.

#### 4.1.3 Mitochondrial injury

Mitochondria are crucial intracellular organelles responsible for energy production and maintenance of cell homeostasis. They are highly enriched in the proximal tubules, serving as a vital source of adenosine triphosphate (ATP) (Galvan et al., 2017). However, mitochondria are susceptible to a variety of intrinsic and environmental stressors, which can render them vulnerable and dysregulated (Tang et al., 2021). A growing body of evidence has shown the connection between mitochondrial dysfunction and CKD progression (Huang et al., 2024). In CKD patients, the uremic toxin *p*-cresol accumulates. Research has reported that *p*-cresol suppresses mitochondrial respiration and mitophagy. Specifically, it reduces the activity of mitochondrial complexes I and IV, which are essential for the electron transport chain that drives ATP production (Yoon et al., 2018). Moreover, *p*-cresol disrupts mitochondrial dynamics. It promotes mitochondrial mitofusion, leading to the formation of enlarged, interconnected mitochondrial networks. This process is accompanied by an elevation in the level of cytochrome c oxidase subunit 4 (COX4) (Chiao-Yin et al., 2017). The changes in mitochondrial structure and the upregulation of COX4 further interfere with normal mitochondrial function, exacerbating mitochondrial dysfunction in the context of CKD (Yoon et al., 2018).

#### 4.1.4 Complications

CKD patients frequently experience complications like uremic pruritus. Protease-activated receptor 2 (PAR-2) plays a key role in mediating itching. In addition to being highly expressed in epidermal keratinocytes of patients with atopic dermatitis (Zhao et al., 2020). PAR-2 is also significantly upregulated in CKD patients with pruritus. These patients exhibit higher protease activity and greater expression level of PAR-2 compared to healthy controls (Moon et al., 2014). Reports have indicated that *p*-cresol is involved in the development of uremic pruritus in CKD patients. When normal human epidermal keratinocytes are exposed to *p*-cresol, both the mRNA and protein expression of PAR-2 are significantly upregulated, and protease activity increases (Kim et al., 2021). Nevertheless, the exact pathogenesis underlying uremic pruritus induced by *p*-cresol still requires further investigation.

Diabetes mellitus is another prevalent complication among CKD patients. Approximately 40% of diabetes mellitus patients progress to diabetic nephropathy (Oladi-Ghadikolaei et al., 2023). In these patients, the uremic toxin *p*-cresol accumulates significantly. Evidence suggests that *p*-cresol may contribute to the development of nephropathy in diabetic individuals (Oladi-Ghadikolaei et al., 2023; Zhu et al., 2023). Emerging evidence suggests that the level of *p*-cresol can potentially serve as a predictive marker for the progression of kidney failure in patients with diabetic nephropathy. This underlines the importance of understanding the role of *p*-cresol in the context of diabetic nephropathy, a prevalent comorbidity among CKD patients.

### 4.2 Impacts of *p*-cresol on the colon

**FIGURE 4:**
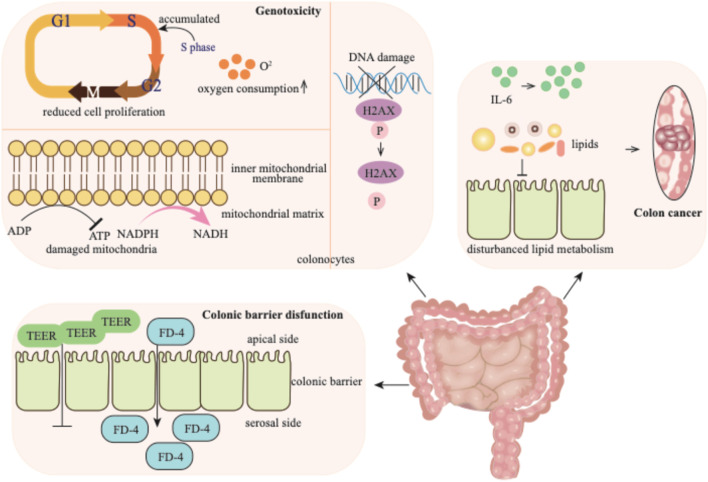
*p*-Cresol exhibits detrimental effects on the colon: it elicits genotoxic injury in colonocytes, disrupts the colonic barrier function, and ultimately drives malignant transformation toward colorectal carcinogenesis. TEER: transepithelial electrical resistance; FD-4: fluorescein isothiocyanate-dextran.

Numerous studies have reported the association between *p*-cresol and colon-related diseases (Blachier and Andriamihaja, 2022). *p*-Cresol predominantly exhibits genotoxicity to colonocytes, impairing colonic barrier function and, in more severe cases, contributing to the development of colon cancer (Figure 4).

#### 4.2.1 Genotoxicity

*p*-Cresol is generated in the colon and exerts genotoxicity on colonocytes (Andriamihaja et al., 2015). When acting on colonocytes, *p*-cresol can induce diverse outcomes, such as reduced cell proliferation and mitochondrial function, as well as increased DNA damage. Exposure to *p*-cresol causes colonocytes to accumulate in the S-phase of cell cycle, indicating a disruption in cell cycle progression. Concurrently, an elevation in oxygen consumption is observed. The detrimental effect of *p*-cresol on mitochondrial function is manifested by the increased oxidation of mitochondrial NADPH/NADH and decreased ATP content (Andriamihaja et al., 2015; Wong et al., 2016). *p*-Cresol also induces the phosphorylation of the H2AX histone in a dose-dependent manner, indicating its effect on DNA damage (Andriamihaja et al., 2015).

#### 4.2.2 Colonic barrier disfunction

The colonic barrier function is highly sensitive to *p*-cresol. The transepithelial electrical resistance (TEER) level and the flux of fluorescein isothiocyanate-dextran (FD-4) across the monolayer from the apical to the serosal side serve as important indicators, reflecting the integrity and permeability of the epithelial paracellular pathway, respectively (Wong et al., 2016). When the intestinal cell monolayer is exposed to *p*-cresol, the TEER value decreases, while the transport of FD-4 across the epithelial monolayer correspondingly increases. These changes clearly indicate that *p*-cresol can disrupt the gut barrier function.

#### 4.2.3 Colon cancer

*p*-Cresol has been reported to have a positive association with colon cancer (Diether and Willing, 2019). In the feces of colorectal cancer patients, *p*-cresol is present at a significantly high level compared to healthy individuals (Alustiza et al., 2023). Multiple factors contribute to the development of colon cancer. The upregulated expression of proinflammatory cytokine IL-6 and the disturbance of lipid metabolism may underlie the mechanism of intestinal tumorigenesis (F. Li et al., 2015). It is likely that *p*-cresol, being present at high levels in colorectal cancer patients, may contribute to this process, potentially by influencing cytokine expression and lipid metabolism. Additionally, due to its significantly elevated levels in the feces of colorectal cancer patients, the level of *p*-cresol has been proposed as a biomarker for the detection of colorectal cancer and pre-malignant lesion (Alustiza et al., 2023).

#### 4.2.4 Impacts of *p*-cresol on the central nervous system

**FIGURE 5:**
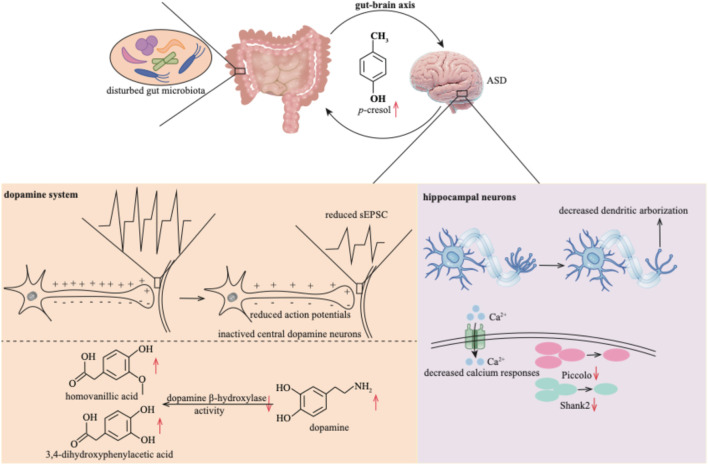
*p*-Cresol exerts neurotoxicity within the central nervous system by establishing a deleterious feedback loop with the gut microbiota. This bidirectional axis disrupts dopaminergic signaling and progressively undermines the structural integrity and functional plasticity of hippocampal neurons. sEPSC: spontaneous excitatory post-synaptic currents.

The microbe-gut-brain axis is pivotal in shaping central nervous system function (Xia et al., 2025), and nowhere is this more evident than in autism spectrum disorder (ASD)—a prevalent neuropsychiatric condition defined by profound social-communicative deficits (Zhuang et al., 2024). Because the gut microbiome is the predominant source of *p*-cresol, sustained overproduction of this metabolite becomes especially deleterious to individuals with ASD (Figure 5). Elevated urinary and fecal *p*-cresol has been consistently documented in ASD patients (Zheng et al., 2022), and mechanistic studies now reveal a bidirectional toxicity: *p*-cresol not only accumulates as a microbial by-product but also re-sculpts the microbial community itself, precipitating autistic-like behaviors (Bermudez-Martin et al., 2021). Consequently, targeted modulation of the gut microbiota is emerging as a promising therapeutic strategy to mitigate ASD symptomatology (Turriziani et al., 2022).

In an open-field environment, *p*-cresol also triggers anxiety-like behaviors and affects the locomotor activity of mice with ASD (Pascucci et al., 2020). Studies have indicated elevated neurotransmitter levels in ASD patients, for instance, dopamine (Gevi et al., 2020). However, *p*-cresol attenuates the excitability of central dopamine neurons. This is evidenced by a reduction in the number of evoked action potentials, as well as reduced amplitudes and frequencies of miniature spontaneous excitatory post-synaptic currents (sEPSC) (Bermudez-Martin et al., 2021). *p*-Cresol further inhibits dopamine *β*-hydroxylase activity (Shaw, 2024) and disrupts dopamine metabolism, altering the levels of dopamine, homovanillic acid, and 3,4-dihydroxyphenylacetic acid (Pascucci et al., 2020). Ultimately, these disruptions to the dopamine system lead to biochemical damage to brain cells (Shaw, 2024).

Beyond its impact on the dopamine system, *p*-cresol impairs the function of hippocampal neurons. It does so by decreasing dendritic arborization, decreasing the number of Piccolo and Shank2 proteins, and blunting the intracellular calcium response in hippocampal neurons (Guzman-Salas et al., 2022). The underlying mechanisms through which *p*-cresol contributes to the development of ASD remain to be further explored.

### 4.3 Impacts of *p*-cresol on the heart

**FIGURE 6:**
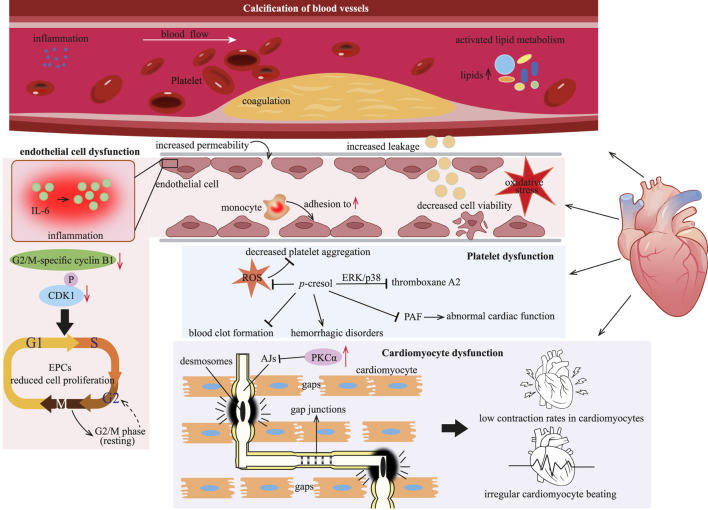
*p*-Cresol inflicts cardiac injury by driving vascular calcification, provoking endothelial dysfunction, suppressing proliferation of EPCs, and ultimately precipitating cardiomyocyte malfunction. EPCs: endothelial progenitor cells; PKCα: protein kinase Cα; ERK: extracellular signal-regulated kinase; PAF: platelet activating factor; ROS: reactive oxygen species.

*p*-Cresol is regarded as detrimental to the cardiovascular system. Specifically, it exerts adverse effects on multiple critical components of this system, including driving arterial media calcification, disrupting the viability and barrier function of cardiovascular endothelial cells, impairing the contractile performance of cardiomyocytes, as well as suppressing the activation and aggregation of platelets. These multifaceted toxic effects are further illustrated in Figure 6.

#### 4.3.1 Calcification of blood vessels

Cardiovascular disease (CVD) is particularly common among CKD patients. In these patients, the accumulation of uremic toxins, such as *p*-cresol, exacerbates the risk of developing CVD. Arterial media calcification is a major life-threatening factor contributing to cardiovascular mortality in CKD patients (Opdebeeck et al., 2020). Inflammation and coagulation within the arterial wall are closely associated with the calcification induced by urinary toxins (Opdebeeck et al., 2019). Both free *p*-cresol and its circulating form, *p*-cresyl sulfate (Figures 1A,B), are urinary toxins. They are related to a higher incidence of cardiovascular events and functions as risk predictors of CVD in hemodialysis patients (Lin et al., 2010; Meijers et al., 2010). Exposure to *p*-cresol, either in the free state or as *p*-cresyl sulfate, initiates calcification in the aorta and peripheral arteries by activating inflammation, coagulation and lipid metabolism pathways (Opdebeeck et al., 2020; Opdebeeck et al., 2019).

#### 4.3.2 Cardiovascular endothelial cell dysfunction

The vascular endothelium plays a crucial role in maintaining the homeostasis of the cardiovascular system. *p*-Cresol has been demonstrated to have a negative effect on the endothelial cell, contributing to endothelial dysfunction (Guerrero et al., 2020). Endothelial dysfunction is considered a key factor triggering the onset and progression of CVD (Opdebeeck et al., 2020). When endothelial cells are exposed to *p*-cresol over an extended period, vascular permeability and leakage increase, and endothelial cell viability declines (Mankhong et al., 2024). This damage to the endothelial cells further enables *p*-cresol to promote monocyte adhesion to the vessel endothelium. As a result, it induces IL-6 production, thereby eliciting endothelial inflammatory responses (Mankhong et al., 2024).

Impaired endothelial regeneration is another factor contributing to the incidence of CVD. A decreased number of endothelial progenitor cells (EPCs) has been repeatedly shown to be involved in the pathogenesis of cardiovascular events (Heinisch et al., 2022). *p*-Cresol inhibits the proliferation of EPCs in a concentration-dependent manner by restricting vessel formation and cell migration (Zhu et al., 2012). Specifically, treatment with *p*-cresol results in an extended cell cycle at the G2/M phase of EPCs, as evidenced by decreased levels of G2/M-specific cyclin B1 and phosphorylated CDK1 (Zhu et al., 2012). The reduction in EPCs proliferation further impairs the endothelium’s ability to repair itself, exacerbating endothelial dysfunction and increasing the risk of CVD.

Regarding the underlying toxicological mechanism, *p*-cresol might disrupt the normal redox balance within endothelial cells. Evidently, *p*-cresol could induce oxidative stress, which in turn leads to the various detrimental effects on the endothelium (Pan et al., 2017).

#### 4.3.3 Cardiomyocyte dysfunction

Mounting data indicate the association between *p*-cresol and cardiomyocyte dysfunction. Cardiomyocytes are interconnected through intercalated discs (ICDs), which consist of desmosomes, AJs, and gap junctions (Nielsen et al., 2023). AJs are essential for maintaining the integrity of discs, while gap junctions are responsible for the passage of ions and electric currents between cardiomyocytes (Merkel et al., 2019). The protein kinase Cα (PKCα) signaling pathway is involved in gap junctions, and its activation leads to reduced myocardial contractility and heart failure. *p*-Cresol causes low contraction rates in cardiomyocytes, together with irregular cardiomyocyte beating. This is because *p*-cresol activates PKCα (Velasquez et al., 2018). The activation of PKCα disrupts the normal function of AJs, leading to the formation of functional gaps between cardiomyocytes. As a result, the mechanical and electrical coupling between cardiomyocytes is impaired. Also, the activation of PKCα may be the underlying mechanism through which *p*-cresol exerts its negative effect of on cardiomyocytes (Peng et al., 2012; Peng et al., 2013).

#### 4.3.4 Platelet dysfunction

Uremic patients commonly suffer from platelet dysfunction, which in turn contributes to cardiovascular complications in CKD patients. *p*-Cresol is considered to suppress arachidonic acid (AA)-induced platelet aggregation by decreasing reactive oxygen species (ROS) production. The extracellular signal-regulated kinase (ERK) and p38 MAPKs have been reported to regulate platelet aggregation (Chang et al., 2011). *p*-Cresol can inhibit thromboxane A2 production through the ERK/p38 signaling pathway (Chang et al., 2011). *In vitro* experiments have also confirmed the antiplatelet effect of *p*-cresol, as it inhibits the aggregation of rabbit platelet-rich plasma and mice platelet (Chang et al., 2011). Moreover, due to its antiplatelet property, *p*-cresol may potentially suppress blood clot formation and give rise to hemorrhagic disorders, an area that warrants further investigation (Chang et al., 2011).

Given the crucial role of platelets in maintaining cardiovascular health, other factors associated with cardiovascular function also deserve attention. Platelet activating factor (PAF) can be synthesized by cardiomyocytes when they are appropriately stimulated. However, PAF has been demonstrated to have negative inotropic and arrhythmogenic effects on cardiomyocytes (Ajiro et al., 2011). *p*-Cresol, which affects platelet function, also inhibits PAF production (Dou et al., 2002). By doing so, it may disrupt the normal regulatory mechanisms in the cardiovascular system. Specifically, the inhibitory effect of *p*-cresol on PAF production may lead to abnormal cardiac function, thus contributing to the development of CVD (Chawla et al., 2023).

### 4.4 Other impacts of *p*-cresol

**FIGURE 7:**
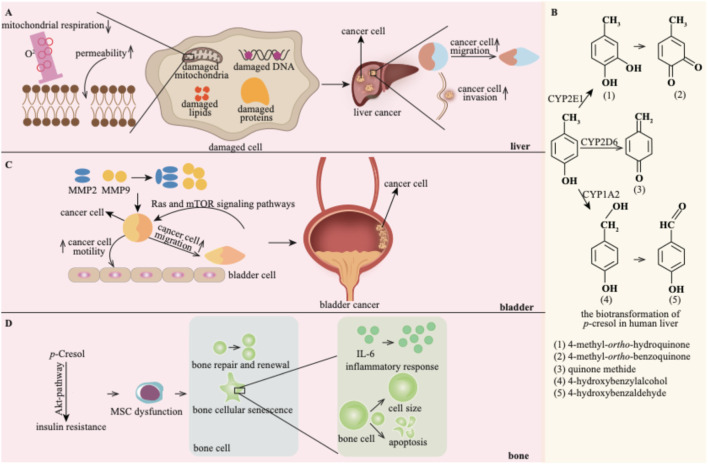
*p*-Cresol exhibits detrimental effects on the liver **(A)**, bladder **(C)** and bone **(D)**. **(B)** shows the biotransformation products of *p*-cresol in the liver (Reprinted with permission from reference (Yan et al., 2005) copyright).

In addition to the detrimental effects of *p*-cresol described above, it also exerts deleterious effects on the liver, bladder, and skeletal muscle (Figure 7).

#### 4.4.1 The harmful effect on the liver

*p*-Cresol exerts hepatotoxic effects in a dose-dependent manner: studies have shown it inhibits liver mitochondrial respiration and increases mitochondrial permeability, thereby identifying mitochondria as a key hepatotoxic target of *p*-cresol (Kitagawa, 2001).

Notably, liver cancer is disproportionately prevalent in hemodialysis patients, a population with impaired p-cresol clearance and chronic systemic accumulation of *p*-cresol (Chen et al., 2023). Given that the liver is the primary organ for *p*-cresol metabolism, the potential tumor-promoting effect of *p*-cresol on the liver warrants in-depth investigation. Accumulated *p*-cresol can facilitate the migration and invasion of liver cancer cells (Chen et al., 2023). In this process, *p*-cresol is oxidized into reactive quinone methide intermediates and 4-methyl-ortho-hydroquinone by cytochrome P450 (Burkina et al., 2018). These reactive biotransformation products of *p*-cresol may damage cellular DNA, proteins, and lipids, leading to hepatic toxicity. Thus, the biotransformation of *p*-cresol might be the underlying toxic mechanism (Yan et al., 2005).

#### 4.4.2 The harmful effect on the bladder

*p*-Cresol has been reported to promote the progression of bladder cancer (Chen et al., 2023). Matrix Metalloproteinases (MMPs) play crucial roles in cancer cell proliferation, migration, and metastasis. *p*-Cresol promotes the motility and migration of bladder cancer cells by upregulating the expression of MMP2 and MMP-9 (Hsu et al., 2019). Additionally, the Ras and mTOR signaling pathways are implicated in the invasion and migration of bladder carcinoma cells induced by *p*-cresol (Hsu et al., 2019).

#### 4.4.3 The harmful effect on the bone

Bone dysfunction represents a newly identified risk factor for CKD patients and may contribute to the incidence of CVD (Fujii et al., 2018). *p*-Cresol is involved in the progression of bone disorders. Bone marrow-derived mesenchymal stem cells (MSCs) circulate into ischemia sites for tissue repair and revascularization. *In vitro* and *in vivo* experiments have demonstrated the functional incompetence of MSCs under uremic conditions, with *p*-cresol being a major uremic toxin (Noh et al., 2014; Noh et al., 2012). *p*-Cresol can induce Akt-pathway-selective insulin resistance, a common occurrence in CKD patients. This, in turn, leads to MSC dysfunction (Noh et al., 2014). MSC dysfunction can disrupt the normal process of bone repair and renewal. Moreover, *p*-cresol is also strongly correlated with bone cellular senescence. Specifically, *p*-cresol increases cell size, stimulates inflammatory responses within cells, disrupts the cell cycle, and concomitantly decreases the cell proliferation capacity (Zayed and Iohara, 2020).

### 4.5 Strategies for removing *p*-cresol

Numerous studies have explored multiple strategies to reduce *p*-cresol concentration. Clinically, hemodialysis, fractionated plasma separation and adsorption (FPSA) (Zare et al., 2022) and hemodiafiltration with endogenous reinfusion (Lu et al., 2024) are used to remove uremic retention solutes. However, *p*-cresol is difficult to remove due to its high protein-binding characteristic (Shen et al., 2020).

*p*-Cresol is a bacteriostatic metabolic by-product produced by gut microbiota with fermentative activity, such as *C. difficile* (Marshall et al., 2023; Passmore et al., 2018). Its production disrupts the balance of gut microbiota, leading to intestinal dysbiosis and facilitating the colonization of *C. difficile* (Marshall et al., 2023). Since an imbalanced gut microbiota is a major driver of increased *p*-cresol production, preventing the colonization of *C. difficile* can suppress the production and toxicity of *p*-cresol, thus maintaining the balance of the intestinal microbiota (Harrison et al., 2022; Mlynarska et al., 2024). Probiotics and prebiotics are promising therapeutic approaches to modulate the microbiota and reduce *p*-cresol production in the CKD patients (Vacca et al., 2021). Synbiotics (SYN), which combine the properties of probiotics and prebiotics, can normalize the intestinal environment and lower serum *p*-cresol concentrations in hemodialysis patients (Vacca et al., 2021). Given that a high plasma level of *p*-cresol in the early stage of CKD is a predictor of progression to end-stage renal disease (ESRD), SYN may also be a possible tool to delay CKD progression towards ESRD (Guida et al., 2014).

Diet also plays a crucial role in *p*-cresol metabolism. Supplementing the diet with protein leads to an increase in *p*-cresol concentration in the plasma (James et al., 2024). Therefore, reducing protein consumption can effectively decrease *p*-cresol levels. Lower protein intake, recommended at 0.6–0.8 g/kg/day, is beneficial for slowing down the deterioration of CKD (Valim et al., 2022). In addition to protein control, dietary polyphenols, such as condensed (proanthocyanidins) or hydrolyzable (ellagitannins and gallotannins) tannins, are effective interventions for reducing protein metabolites (Gasaly and Gotteland, 2022). The administration of tannins can modify the microbiota composition and inhibit the growth of bacterial populations that produce these metabolites (Gasaly and Gotteland, 2022). Although most relevant studies have been conducted on farm animals, the evaluation of the impact of polyphenol on the production of amino acid-derived bacterial metabolites is currently in the preliminarily *in vitro* research stage. The urinary excretion of *p*-cresol decreases after proanthocyanidins administration (Jacobs et al., 2012; Yamakoshi et al., 2009). Polyphenol extracts containing proanthocyanidin have been shown to prevent colonic epithelial cell dysfunction induced by *p*-cresol (Wong et al., 2016). Additionally, a supplementary diet with epigallocatechin gallate (EGCG) from green tea has also been reported to reduce *p*-cresol production in the colon (Unno et al., 2014).

To further enhance *p*-cresol clearance, the addition of activated charcoal to the dialysate has been proposed. This approach can significantly enhance the clearance of protein-bound toxins like *p*-cresol, while leaving the clearance of urea unchanged (Meyer et al., 2007). Subsequently, an oral adsorbent made of active charcoal has been developed to non-specifically bind to uremic toxins (Glorieux and Tattersall, 2015). This is especially suitable for patients who have declined dialysis. For instance, patiromer has been proved to effectively bind to non-adsorbed potassium (Weir et al., 2015). Another oral carbon adsorbent is AST-120, which consists of porous carbon particles and is used to reduce *p*-cresol in the gastrointestinal tract (Sato et al., 2020). AST-120 functions by eliminating reactive oxygen species, thereby helping to reduce inflammation, slow renal function deterioration, mitigate vascular calcification, and improve endothelial function and bone metabolism (Liu et al., 2018).

### 4.6 Utilization of *p*-cresol as a biomarker

*p*-Cresol is generated in the colon by bacteria degrading tyrosine and phenylalanine, and is then excreted in the urine in the form of *p*-cresyl sulfate (Vijayasarathy et al., 2020). The concentration of *p*-cresol is typically associated with diet and colorectal diseases (Al Hinai et al., 2019). By measuring the *p*-cresyl sulfate production in the urine of populations with normal kidney function, it has been discovered that vegetarians excrete 62% less *p*-cresyl sulfate compared to omnivores. Notably, vegetarians have a 69% higher fiber intake and a 25% lower protein intake (Patel et al., 2012). Diet, by influencing *p*-cresol production, may also impact the risk of developing colorectal diseases.

Through *in vitro* fecal culture and cell experiments, *p*-cresol in fermentation supernatants has been found to exhibit genotoxicity effects on colonocytes. It increases DNA damage in colonocytes in a dose-dependent manner (Al Hinai et al., 2019). When fecal samples from individuals with colorectal cancer were tested, *p*-cresol is found to be significantly more abundant in cancer samples compared to other volatile organic compounds. As a result, *p*-cresol has been proposed as a promising biomarker for pre-malignant lesions of the intestine (Alustiza et al., 2023). Additionally, *p*-cresol serves as a useful biomarker for gastric cancer, facilitating the early diagnosis of this disease (Shi et al., 2019).

Beyond its association with clinical diseases, the blood concentration of *p*-cresol in forensic autopsy cases holds diagnostic significance (Ikematsu et al., 2018). Although it does not provide direct information regarding the causes of death, in some forensic autopsy cases involving certain diseases, abnormally high *p*-cresol levels have been detected. These cases include those with atherosclerosis in the basilar or renal arteries, or stenosis in the coronary artery. Thus, the concentration of *p*-cresol can potentially provide valuable information about the antemortem physical conditions of the deceased (Ikematsu et al., 2018).

### 4.7 The potential beneficial properties of *p*-cresol

**FIGURE 8:**
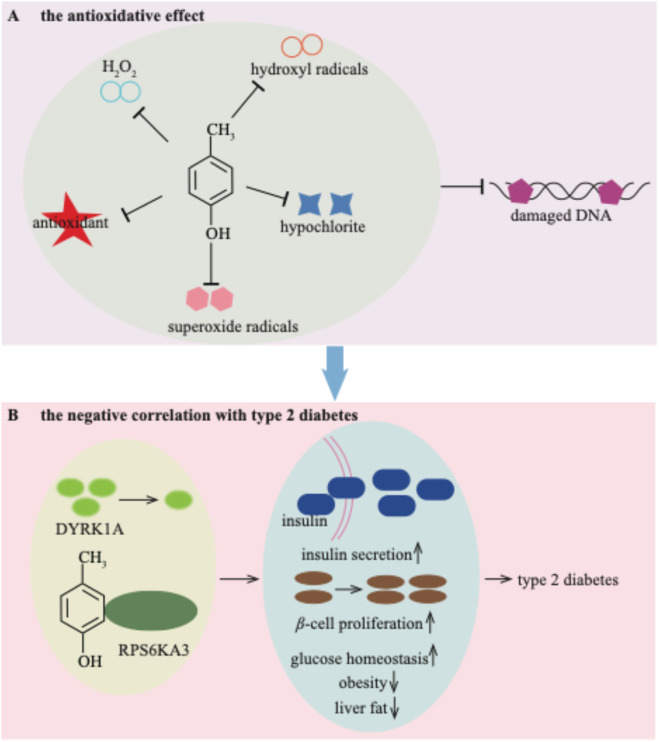
The potential beneficial properties of *p*-cresol for the antioxidative effect **(A)** and negative correlation with type 2 diabetes **(B)**.

Until now, the majority of studies have concentrated on the deleterious effects of *p*-cresol on human health. However, *p*-cresol may possess protective potential. Researchers have reported an antioxidative effect of *p*-cresol on low-density lipoprotein *in vitro* (Ujhelyi et al., 2006a; 2006b). Additionally, *p*-cresol has been proved to have scavenging properties against H_2_O_2_, hypochlorite, superoxide radicals, and hydroxyl radicals. These properties enable it to prevent ROS-induced DNA breaks (Figure 8A; Yeung et al., 2002). Given that oxidative stress is closely associated with the pathogenesis of various metabolic diseases, including type 2 diabetes (Jiawen et al., 2023), the antioxidative properties of *p*-cresol may play a role in its beneficial effects on glucose metabolism.

The serum concentration of *p*-cresol exhibits a negative correlation with type 2 diabetes (Brial et al., 2020). A low dose of *p*-cresol helps to improve glucose homeostasis, reducing obesity and liver fat. Simultaneously, it enhances insulin secretion and *β*-cell function in patients with type 2 diabetes (Brial et al., 2020). The downregulated kinase DYRK1A by *p*-cresol may represent a therapeutic target involved in these mediating effects (Brial et al., 2020). Recently, *p*-cresol has also been shown to stimulate glucose-induced insulin secretion (Brial et al., 2024). Specifically, *p*-cresol binds to ribosomal protein S6 kinase A3 (RPS6KA3), thereby inducing insulin secretion and *β*-cell proliferation (Brial et al., 2024; Figure 8B). The beneficial effects of *p*-cresol on type 2 diabetes provide new therapeutic concepts for addressing insulin deficiency.

## 5 The biochemical activity of *p*-cresol

### 5.1 Function as flavoring substance

*p*-Cresol exhibits a “barny, cowy, medicinal” odor (Kilcawley et al., 2018). Despite being classified as toxic, it has been detected as the off-odor-causing substance in numerous foods (Oshiro et al., 2020). Guangxi fermented bamboo shoot (GFBS), a highly popular food in China, is renowned for its significant health benefits and unique aroma (Li et al., 2022). It has been described as having a “fermented, pungent and sour” aroma, with *p*-cresol identified as the key aroma-active compound (Li et al., 2022).

Cheese is a fermented milk-sourced product (Mayo et al., 2021). As bacteria degrade the constituents of milk, cheese exhibits a characteristic “cowy-barny, earthy” flavor (Suriyaphan et al., 2001). *p*-Cresol has been identified as a flavoring compound in cheese (Oshiro et al., 2020). The addition of *p*-cresol (≥100 ppb) to cheese can intensify the “cowy” flavor notes (Suriyaphan et al., 2001). Moreover, *p*-cresol is responsible for the “barnyard aroma” in bovine milk (Faulkner et al., 2018).

In addition to these food items, liquor, including Chinese liquors, gives off a whiskey-like smoky flavor (Hayes et al., 2020). *p*-Cresol has been recognized as a contributor to the off-odor in aroma-type liquors (Du et al., 2017; Hayes et al., 2020). The theaflavins in black tea have been shown to inhibit the formation of off-odorants in liquors, including *p*-cresol (UENO et al., 2006). This finding may provide an approach for reducing off-odors. Besides liquors, *p*-cresol is also highly concentrated in brown beers, and its level further increases as the beer ages (Scholtes et al., 2014).

Despite the toxicity, *p*-cresol is listed as a fragrance ingredient in the Chinese Standard for the Use of Food Additives (GB2760-2024) (National Food Safety Standard: Standard for the Use of Food Additives, 2024) and in the Research Institute for Fragrance Materials database (http://fragrancematerialsafetyresource.elsevier.com) (Api et al., 2021). *p*-Cresol has been assessed as safe for human health, with no genotoxic effects observed at levels lower than 50 mg/kg/day (Api et al., 2021).

Moving beyond the food and beverage domain, *p*-cresol also contributes to the scent of various plants, playing roles such as providing olfactory cues, visual signals, or attracting pollinators. For instance, it is present in the floral scent of sapromyophilous *Stemona* species (Chen et al., 2017), the scent of *Narcissus viridiflorus* (Losch and Weigend, 2024), and gives rise to the distinct ‘horse urine-like’ odor in *Gastrodia elata* Bl. (Li et al., 2025).

### 5.2 Function as an estrus-specific volatile compound

*p*-Cresol is an estrus-specific volatile compound in livestock species (Karthikeyan et al., 2014). It can be detected in the urine, vaginal mucus and feces of buffaloes during their estrus period (Karthikeyan et al., 2013; Rajanarayanan and Archunan, 2011). Male buffaloes are attracted to the *p*-cresol secreted by female buffalo and exhibit repeated flehmen behavior (Rajanarayanan and Archunan, 2011). Furthermore, a behavior assay has shown that bulls also exhibit repeated flehmen and a higher frequency of mounting behavior when exposed to *p*-cresol (Karthikeyan et al., 2013). Interestingly, a pheromone-based kit has been developed for non-invasive estrus detection in buffaloes (Muthukumar et al., 2018).

Similar to the situation in buffaloes, *p*-cresol also plays an important role in the estrus of mares (Karthikeyan et al., 2014). The concentration of *p*-cresol starts to rise, reaching its peak 1 day prior to ovulation. Subsequently, after ovulation takes place, the *p*-cresol concentration drops back to the basal level. Notably, the concentration of *p*-cresol demonstrates reproducible temporal variations in relation to the time of ovulation (Mozuraitis et al., 2012). When compared to other compounds, stallions show a greater preference to *p*-cresol and exhibit a high erection level (Buda et al., 2012). As a horse sex pheromone, the non-invasive determination of *p*-cresol can indicate the ovulation time, thus helping to reduce both time and costs (Mozuraitis et al., 2012).

In the animal-kingdom, *p*-cresol has a different effect on rats. *p*-Cresol in male rat urine exerts an attractive effect on female rats (Osada et al., 2009). This shows that the role of *p*-cresol in different animals can vary greatly.

Mosquito species are considered as important vectors for numerous diseases globally (Meier et al., 2025). *p*-Cresol shows a double effect on the oviposition behavior of mosquito species (Mulatier et al., 2022). At low concentrations, it serves as an oviposition attractant for gravid mosquitoes, as seen in species like *Aedes triseriatus*, *Culex quinquefasciatus* and *Culex tarsalis* (Zhu et al., 2013). Conversely, at high concentrations, it acts as a repellent for gravid mosquitos, such as *Ae. Albopictus* and *Aedes aegypti* (Afify and Galizia, 2014).

*p*-Cresol is also a key odorant from pig production. Pig production is an important part of modern animal husbandry. *p*-Cresol’s presence in pig-related environments indicates its potential significance in this field. However, compared with its well-studied functions in other animals, the function of this key odorant in pig production remains to be further investigated (Hansen et al., 2018).

### 5.3 Function as a signal molecule

In the animal kingdom, volatiles serve as scent-marks that convey information about an animal’s health, reproductive status, and territorial ownership (Soso and Koziel, 2017). *p*-Cresol is commonly detected in the urine of numerous mammals. For instance, it is present in the urine of lions (*Panthera leo*), where it may play a role in lion reproduction (Soso and Koziel, 2017). These volatiles, including *p*-cresol, released from the urine can potentially interact with other organisms in the environment, such as blood-sucking insects.

As a signal molecule, *p*-cresol attracts blood-sucking insects, being crucial for their nutrition and reproduction (Baldacchino et al., 2014). Aged horse urine, which contains high levels of *p*-cresol, elicits strong electroantennogram responses in horseflies and lures them, thereby facilitating their nutrition-seeking and reproductive activities (Baldacchino et al., 2014). Additionally, after *p*-cresol secreted by immature female rats and released into the environment, *p*-cresol serves as a signal molecule that enables the Asian tiger mosquito, *Aedes albopictus,* to locate their hosts (Diaz-Santiz et al., 2020).

*p*-Cresol is a component present in human sweat (Haag et al., 2023). It has the ability to evoke dose-dependent electroantennography responses in the anthropophilic malaria vector mosquito, *Anopheles gambiae* Giles (Biessmann et al., 2010; Hallem et al., 2004).

## 6 Conclusion and future outlooks

*p*-Cresol is a ubiquitous substance in the environment, as well as in plants and animals. Here, we primarily focus on its effects on humans and animals.

This review synthesizes evidence that *p*-cresol exerts dualistic biological effects: on one hand, it acts as a potent uremic toxin that accumulates in the human body—particularly in patients with CKD—due to its high affinity for binding to plasma proteins. In light of this, the analysis further outlines multiple strategies proposed for *p*-cresol removal, including microbiota modulation, dietary optimization, and activated charcoal adsorption. To effectively eliminate the *p*-cresol-protein complex, a comprehensive understanding of the physical properties of these proteins and their binding mechanisms with *p*-cresol is essential. Novel technologies targeting this complex—such as Proteolysis Targeting Chimera (PROTAC), which promotes proteolysis—may offer promising solutions for enhanced *p*-cresol removal. Notably, polyphenol extracts have shown effectiveness in modulating microbiota composition, thereby reducing the production of protein metabolites (including *p*-cresol); given that many traditional medicines are rich in polyphenols, developing traditional Chinese medicine products could provide additional auxiliary treatment options for CKD patients.

On the other hand, *p*-cresol also possesses under-appreciated beneficial properties, such as antioxidant activity and the ability to modulate glucose homeostasis. However, further research is needed to fully explore and validate these potential benefits. Regardless of its dual effects, the proper and regulated use of *p*-cresol is of critical importance; striving to maximize its beneficial roles while minimizing potential harms represents the most rational approach. The findings summarized in this review are expected to contribute to the safe management and rational application of *p*-cresol.

Despite these insights, several limitations of this review must be acknowledged. First, most mechanistic studies on *p*-cresol have been conducted *in vitro* or in animal models, leaving the translational relevance of these findings to human CKD populations uncertain. Second, the analysis focuses heavily on *p*-cresol’s effects in humans and animals but only briefly mentions its roles in plants, leading to incomplete coverage of its biological functions across different biological kingdoms—key studies on plant-*p*-cresol interactions (e.g., its role in plant defense or symbiosis) were not systematically included. Third, the potential beneficial roles of *p*-cresol at physiological concentrations are supported by limited and scattered data, lacking systematic verification. Addressing these knowledge gaps through well-controlled clinical studies, cross-species comparative analyses, and structure–function investigations will be essential before any evidence-based therapeutic or regulatory recommendations for *p*-cresol can be confidently advanced.

## References

[B1] Afify A. Galizia C. G. (2014). Gravid females of the mosquito *Aedes aegypti* avoid oviposition on m-cresol in the presence of the deterrent isomer p-cresol. Parasites and Vectors 7, 315. 10.1186/1756-3305-7-315 25008201 PMC4101705

[B2] Ajiro Y. Saegusa N. Giles W. R. Stafforini D. M. Spitzer K. W. (2011). Platelet-activating factor stimulates sodium-hydrogen exchange in ventricular myocytes. Am. J. Physiol. Heart Circ. Physiol. 301 (6), H2395–H2401. 10.1152/ajpheart.00607.2011 21949111 PMC3233817

[B3] Al Hinai E. A. Kullamethee P. Rowland I. R. Swann J. Walton G. E. Commane D. M. (2019). Modelling the role of microbial p-cresol in colorectal genotoxicity. Gut Microbes 10 (3), 398–411. 10.1080/19490976.2018.1534514 30359553 PMC6546321

[B4] Alustiza M. Ripoll L. Canals A. Murcia O. Martinez-Roca A. Garcia-Heredia A. (2023). A novel non-invasive colorectal cancer diagnostic method: volatile organic compounds as biomarkers. Clin. Chim. Acta 542, 117273. 10.1016/j.cca.2023.117273 36863694

[B5] Andriamihaja M. Lan A. Beaumont M. Audebert M. Wong X. Yamada K. (2015). The deleterious metabolic and genotoxic effects of the bacterial metabolite p-cresol on colonic epithelial cells. Free Radic. Biol. Med. 85, 219–227. 10.1016/j.freeradbiomed.2015.04.004 25881551

[B6] Api A. M. Belsito D. Botelho D. Bruze M. Burton G. A. Jr. Buschmann J. (2021). RIFM fragrance ingredient safety assessment, p-cresol, CAS registry number 106-44-5. Food Chem. Toxicol. 158 (Suppl. 1), 112631. 10.1016/j.fct.2021.112631 34688704

[B7] Arts J. J. Mahlandt E. K. Gronloh M. L. Schimmel L. Noordstra I. Gordon E. (2021). Endothelial junctional membrane protrusions serve as hotspots for neutrophil transmigration. Elife 10, e66074. 10.7554/eLife.66074 34431475 PMC8437435

[B8] Baldacchino F. Manon S. Puech L. Buatois B. Dormont L. Jay-Robert P. (2014). Olfactory and behavioural responses of tabanid horseflies to octenol, phenols and aged horse urine. Med. Vet. Entomol. 28 (2), 201–209. 10.1111/mve.12038 24188243

[B9] Bermudez-Martin P. Becker J. A. J. Caramello N. Fernandez S. P. Costa-Campos R. Canaguier J. (2021). The microbial metabolite p-Cresol induces autistic-like behaviors in mice by remodeling the gut microbiota. Microbiome 9 (1), 157. 10.1186/s40168-021-01103-z 34238386 PMC8268286

[B10] Biessmann H. Andronopoulou E. Biessmann M. R. Douris V. Dimitratos S. D. Eliopoulos E. (2010). The *Anopheles gambiae* odorant binding protein 1 (AgamOBP1) mediates indole recognition in the antennae of female mosquitoes. PLoS One 5 (3), e9471. 10.1371/journal.pone.0009471 20208991 PMC2830424

[B11] Blachier F. Andriamihaja M. (2022). Effects of the L-tyrosine-derived bacterial metabolite p-cresol on colonic and peripheral cells. Amino Acids 54 (3), 325–338. 10.1007/s00726-021-03064-x 34468872

[B12] Brial F. Alzaid F. Sonomura K. Kamatani Y. Meneyrol K. Le Lay A. (2020). The natural metabolite 4-Cresol improves glucose homeostasis and enhances beta-cell function. Cell Rep. 30 (7), 2306–2320. 10.1016/j.celrep.2020.01.066 32075738

[B13] Brial F. Puel G. Gonzalez L. Russick J. Auld D. Lathrop M. (2024). Stimulation of insulin secretion induced by low 4-cresol dose involves the RPS6KA3 signalling pathway. PloS One 19 (10), e0310370. 10.1371/journal.pone.0310370 39446839 PMC11500888

[B14] Brocca A. Virzi G. M. de Cal M. Cantaluppi V. Ronco C. (2013). Cytotoxic effects of p-cresol in renal epithelial tubular cells. Blood Purif. 36 (3-4), 219–225. 10.1159/000356370 24496194

[B15] Buda V. Mozuraitis R. Kutra J. Borg-Karlson A. K. (2012). p-Cresol: a sex pheromone component identified from the estrous urine of mares. J. Chem. Ecol. 38 (7), 811–813. 10.1007/s10886-012-0138-2 22592336

[B16] Burkina V. Sakalli S. Zlabek V. Zamaratskaia G. (2018). CYP1A1 activity in rainbow trout is inhibited by the environmental pollutant p-cresol. Environ. Toxicol. Pharmacol. 62, 199–202. 10.1016/j.etap.2018.07.013 30077163

[B17] Campillo S. Bohorquez L. Gutierrez-Calabres E. Garcia-Ayuso D. Miguel V. Griera M. (2022). Indoxyl sulfate- and P-cresol-induced monocyte adhesion and migration is mediated by integrin-linked kinase-dependent podosome formation. Exp. Mol. Med. 54 (3), 226–238. 10.1038/s12276-022-00738-8 35246616 PMC8980039

[B18] Cerini C. Dou L. Anfosso F. Sabatier F. Moal V. Glorieux G. (2004). P-cresol, a uremic retention solute, alters the endothelial barrier function *in vitro* . Thrombosis Haemostasis 92 (1), 140–150. 10.1160/TH03-07-0491 15213855

[B19] Chang M. C. Wang T. M. Yeung S. Y. Jeng P. Y. Liao C. H. Lin T. Y. (2011). Antiplatelet effect by p-cresol, a uremic and environmental toxicant, is related to inhibition of reactive oxygen species, ERK/p38 signaling and thromboxane A2 production. Atherosclerosis 219 (2), 559–565. 10.1016/j.atherosclerosis.2011.09.031 21993412

[B20] Chawla N. Shah H. Huynh K. Braun A. Wollocko H. Shah N. C. (2023). The role of platelet-activating factor and magnesium in obstetrics and gynecology: is there crosstalk between pre-eclampsia, clinical hypertension, and HELLP syndrome? Biomedicines 11 (5), 1343. 10.3390/biomedicines11051343 37239015 PMC10216284

[B21] Chen G. Gong W. C. Ge J. Schinnerl J. Wang B. Sun W. B. (2017). Variation in floral characters, particularly floral scent, in sapromyophilous stemona species. J. Integr. Plant Biol. 59 (11), 825–839. 10.1111/jipb.12580 28836349

[B22] Chen X. Xiang F. Cao X. Zou J. Zhang B. Ding X. (2023). Effects of p-cresol, a uremic toxin, on cancer cells. Transl. Cancer Res. 12 (2), 367–374. 10.21037/tcr-22-2042 36915599 PMC10007878

[B23] Chiao-Yin S. Mei-Ling C. Heng-Chih P. Jia-Hung L. Chin-Chan L. (2017). Protein-bound uremic toxins impaired mitochondrial dynamics and functions. Oncotarget 8 (44), 77722–77733. 10.18632/oncotarget.20773 29100420 PMC5652810

[B24] Di Paola R. De A. Izhar R. Abate M. Zappavigna S. Capasso A. (2023). Possible effects of uremic toxins p-Cresol, indoxyl sulfate, p-Cresyl sulfate on the development and progression of Colon cancer in patients with chronic renal failure. Genes (Basel) 14 (6), 1257. 10.3390/genes14061257 37372437 PMC10298729

[B25] Diaz-Santiz E. Rojas J. C. Casas-Martinez M. Cruz-Lopez L. Malo E. A. (2020). Rat volatiles as an attractant source for the Asian tiger mosquito, *Aedes albopictus* . Sci. Rep. 10 (1), 5170. 10.1038/s41598-020-61925-z 32198359 PMC7083917

[B26] Diether N. E. Willing B. P. (2019). Microbial fermentation of dietary protein: an important factor in Diet(-)Microbe(-)Host interaction. Microorganisms 7 (1), 19. 10.3390/microorganisms7010019 30642098 PMC6352118

[B27] Dodd D. Spitzer M. H. Van Treuren W. Merrill B. D. Hryckowian A. J. Higginbottom S. K. (2017). A gut bacterial pathway metabolizes aromatic amino acids into nine circulating metabolites. Nature 551 (7682), 648–652. 10.1038/nature24661 29168502 PMC5850949

[B28] Dou L. Cerini C. Brunet P. Guilianelli C. Moal V. Grau G. (2002). P-cresol, a uremic toxin, decreases endothelial cell response to inflammatory cytokines. Kidney Int. 62 (6), 1999–2009. 10.1046/j.1523-1755.2002.t01-1-00651.x 12427124

[B29] Du H. Liu B. Wang X. Xu Y. (2017). Exploring the microbial origins of p-cresol and its co-occurrence pattern in the Chinese liquor-making process. Int. J. Food Microbiol. 260, 27–35. 10.1016/j.ijfoodmicro.2017.08.016 28843121

[B30] Faulkner H. O'Callaghan T. F. McAuliffe S. Hennessy D. Stanton C. O'Sullivan M. G. (2018). Effect of different forage types on the volatile and sensory properties of bovine milk. J. Dairy Sci. 101 (2), 1034–1047. 10.3168/jds.2017-13141 29224876

[B31] Flynn C. K. Adams J. B. Krajmalnik-Brown R. Khoruts A. Sadowsky M. J. Nirmalkar K. (2025). Review of elevated para-cresol in autism and possible impact on symptoms. Int. J. Mol. Sci. 26 (4), 1513. 10.3390/ijms26041513 40003979 PMC11855632

[B32] Fujii H. Goto S. Fukagawa M. (2018). Role of uremic toxins for kidney, cardiovascular, and bone dysfunction. Toxins 10 (5), 202. 10.3390/toxins10050202 29772660 PMC5983258

[B33] Galvan D. L. Green N. H. Danesh F. R. (2017). The hallmarks of mitochondrial dysfunction in chronic kidney disease. Kidney Int. 92 (5), 1051–1057. 10.1016/j.kint.2017.05.034 28893420 PMC5667560

[B34] Gasaly N. Gotteland M. (2022). Interference of dietary polyphenols with potentially toxic amino acid metabolites derived from the colonic microbiota. Amino Acids 54 (3), 311–324. 10.1007/s00726-021-03034-3 34235577

[B35] Gevi F. Belardo A. Zolla L. (2020). A metabolomics approach to investigate urine levels of neurotransmitters and related metabolites in autistic children. Biochim. Biophys. Acta Mol. Basis Dis. 1866 (10), 165859. 10.1016/j.bbadis.2020.165859 32512190

[B36] Glorieux G. Tattersall J. (2015). Uraemic toxins and new methods to control their accumulation: game changers for the concept of dialysis adequacy. Clin. Kidney J. 8 (4), 353–362. 10.1093/ckj/sfv034 26251699 PMC4515890

[B37] Glorieux G. Gryp T. Perna A. (2020). Gut-derived metabolites and their role in immune dysfunction in chronic kidney disease. Toxins 12 (4), 245. 10.3390/toxins12040245 32290429 PMC7232434

[B38] Gryp T. Vanholder R. Vaneechoutte M. Glorieux G. (2017). p-Cresyl sulfate. Toxins (Basel) 9 (2), 52. 10.3390/toxins9020052 28146081 PMC5331431

[B39] Guerrero F. Carmona A. Obrero T. Jimenez M. J. Soriano S. Moreno J. A. (2020). Role of endothelial microvesicles released by p-cresol on endothelial dysfunction. Sci. Rep. 10 (1), 10657. 10.1038/s41598-020-67574-6 32606426 PMC7326964

[B40] Guida B. Germano R. Trio R. Russo D. Memoli B. Grumetto L. (2014). Effect of short-term synbiotic treatment on plasma p-cresol levels in patients with chronic renal failure: a randomized clinical trial. Nutr. Metabolism, Cardiovasc. Dis. 24 (9), 1043–1049. 10.1016/j.numecd.2014.04.007 24929795

[B41] Guzman-Salas S. Weber A. Malci A. Lin X. Herrera-Molina R. Cerpa W. (2022). The metabolite p-cresol impairs dendritic development, synaptogenesis, and synapse function in hippocampal neurons: implications for autism spectrum disorder. J. Neurochem. 161 (4), 335–349. 10.1111/jnc.15604 35257373

[B42] Haag F. Frey T. Hoffmann S. Kreissl J. Stein J. Kobal G. (2023). The multi-faceted food odorant 4-methylphenol selectively activates evolutionary conserved receptor OR9Q2. Food Chem. 426, 136492. 10.1016/j.foodchem.2023.136492 37295052

[B43] Hallem E. A. Fox A. N. Zwiebel L. J. Carlson J. R. (2004). Olfaction: mosquito receptor for human-sweat odorant. Nature 427 (6971), 212–213. 10.1038/427212a 14724626

[B44] Hansen M. J. Kasper P. L. Adamsen A. P. S. Feilberg A. (2018). Key odorants from pig production based on improved measurements of odor threshold values combining olfactometry and proton-transfer-reaction mass spectrometry (PTR-MS). Sensors (Basel) 18 (3), 788. 10.3390/s18030788 29509664 PMC5876742

[B45] Harrison M. A. Kaur H. Wren B. W. Dawson L. F. (2021). Production of p-cresol by decarboxylation of p-HPA by all five lineages of Clostridioides difficile provides a growth advantage. Front. Cell Infect. Microbiol. 11, 757599. 10.3389/fcimb.2021.757599 34778108 PMC8588808

[B46] Harrison M. A. Strahl H. Dawson L. F. (2022). Regulation of para-cresol production in Clostridioides difficile. Curr. Opin. Microbiol. 65, 131–137. 10.1016/j.mib.2021.11.005 34856509

[B47] Hayes P. E. Luong J. H. T. Gilchrist E. S. Buzid A. Glennon J. D. (2020). Profiling of phenolic flavorings using core-shell reversed-phase liquid chromatography with electrochemical detection at a boron-doped diamond electrode. J. Chromatogr. A 1612, 460649. 10.1016/j.chroma.2019.460649 31708221

[B48] Heinisch P. P. Bello C. Emmert M. Y. Carrel T. Dressen M. Horer J. (2022). Endothelial progenitor cells as biomarkers of cardiovascular pathologies: a narrative review. Cells 11 (10), 1678. 10.3390/cells11101678 35626716 PMC9139418

[B49] Hsu Y. H. Huang H. P. Chang H. R. (2019). The uremic toxin p-cresol promotes the invasion and migration on carcinoma cells *via* ras and mTOR signaling. Toxicol. Vitro 58, 126–131. 10.1016/j.tiv.2019.03.029 30910523

[B50] Huang J. Hao J. Wang P. Xu Y. (2024). The role of mitochondrial dysfunction in CKD-related vascular calcification: from mechanisms to therapeutics. Kidney Int. Rep. 9 (9), 2596–2607. 10.1016/j.ekir.2024.05.005 39291213 PMC11403042

[B51] Ikematsu N. Kashiwagi M. Hara K. Waters B. Matsusue A. Takayama M. (2018). Diagnostic meaning of blood p-cresol concentration in forensic autopsy cases. Leg. Med. Tokyo, Jpn. 34, 27–35. 10.1016/j.legalmed.2018.08.003 30103078

[B52] Jacobs D. M. Fuhrmann J. C. van Dorsten F. A. Rein D. Peters S. van Velzen E. J. (2012). Impact of short-term intake of red wine and grape polyphenol extract on the human metabolome. J. Agric. Food Chem. 60 (12), 3078–3085. 10.1021/jf2044247 22372405

[B53] James D. Poveda C. Walton G. E. Elmore J. S. Linden B. Gibson J. (2024). Do high-protein diets have the potential to reduce gut barrier function in a sex-dependent manner? Eur. J. Nutr. 63 (6), 2035–2054. 10.1007/s00394-024-03407-w 38662018 PMC11377480

[B54] Ji M. Du H. Xu Y. (2020). Structural and metabolic performance of p-cresol producing microbiota in different carbon sources. Food Res. Int. 132, 109049. 10.1016/j.foodres.2020.109049 32331677

[B55] Jia-ying Z. Jin L. (2022). Research progress in the detection of volatile phenol. Chin. J. Urban Rural Enterp. Hyg. 37 (09), 62–64. 10.16286/j.1003-5052.2022.09.023

[B56] Jiawen C. Qi W. Ruiyan L. Zhe L. Qizhou J. Fangrong Y. (2023). The role of sirtuins in the regulatin of oxidative stress during the progress and therapy of type 2 diabetes mellitus. Life Sci. 333, 122187. 10.1016/j.lfs.2023.122187 37858715

[B57] Karthikeyan K. Muniasamy S. SankarGanesh D. Achiraman S. Ramesh Saravanakumar V. Archunan G. (2013). Faecal chemical cues in water buffalo that facilitate estrus detection. Animal Reproduction Sci. 138 (3-4), 163–167. 10.1016/j.anireprosci.2013.02.017 23570909

[B58] Karthikeyan K. Manivannan P. Rajesh D. Muthukumar S. Muralitharan G. Akbarsha M. A. (2014). Identification of p-cresol as an estrus-specific volatile in buffalo saliva: comparative docking analysis of buffalo OBP and beta-lactoglobulin with p-cresol. Zoological Sci. 31 (1), 31–36. 10.2108/zsj.31.31 24410493

[B59] Kawakami K. Makino I. Kato I. Uchida K. Onoue M. (2009). p-Cresol inhibits IL-12 production by murine macrophages stimulated with bacterial immunostimulant. Immunopharmacol. Immunotoxicol. 31 (2), 304–309. 10.1080/08923970802680299 19235606

[B60] Kihara T. Toriuchi K. Aoki H. Kakita H. Yamada Y. Aoyama M. (2021). Interleukin-1β enhances cell adhesion in human endothelial cells *via* microRNA-1914-5p suppression. Biochem. Biophys. Rep. 27, 101046. 10.1016/j.bbrep.2021.101046 34179516 PMC8214032

[B61] Kilcawley K. N. Faulkner H. Clarke H. J. O'Sullivan M. G. Kerry J. P. (2018). Factors influencing the flavour of bovine milk and cheese from grass based *versus* non-grass based milk production systems. Foods 7 (3), 37. 10.3390/foods7030037 29534042 PMC5867552

[B62] Kim S. J. Zhang X. Cho S. B. Kim C. H. Park H. C. Moon S. J. (2021). Uremic solutes of indoxyl sulfate and p-cresol enhance protease-activated receptor-2 expression *in vitro* and *in vivo* in keratinocytes. Hum. Exp. Toxicol. 40 (1), 113–123. 10.1177/0960327120945758 32757783

[B63] Kitagawa A. (2001). Effects of cresols (o-m-and p-isomers) on the bioenergetic system in isolated rat liver mitochondria. Drug Chem. Toxicol. 24 (1), 39–47. 10.1081/dct-100103084 11307633

[B64] Kriek M. Martins F. Challand M. R. Croft A. Roach P. L. (2007). Thiamine biosynthesis in escherichia coli: identification of the intermediate and by-product derived from tyrosine. Angew. Chem. Int. Ed. Engl. 46 (48), 9223–9226. 10.1002/anie.200702554 17969213

[B65] Krogt J. M. V. D. Meulen I. J. V. D. Buul J. D. V. (2023). Spatiotemporal regulation of Rho GTPase signaling during endothelial barrier remodeling. Curr. Opin. physiology 34, 100676. 10.1016/j.cophys.2023.100676 PMC10398679 37547802

[B66] Lee S. H. Cho W. J. Najy A. J. Saliganan A.-D. Pham T. Rakowski J. (2021). p62/SQSTM1-induced caspase-8 aggresomes are essential for ionizing radiation-mediated apoptosis. Cell death and Dis. 12 (11), 997. 10.1038/s41419-021-04301-7 34697296 PMC8546074

[B67] Li F. Yang X. W. Krausz K. W. Nichols R. G. Xu W. Patterson A. D. (2015). Modulation of colon cancer by nutmeg. J. Proteome Res. 14 (4), 1937–1946. 10.1021/pr5013152 25712450 PMC6334305

[B68] Li S. Tian Y. Sun M. Liu J. Bai Y. Liu X. (2022). Characterization of key aroma compounds in fermented bamboo shoots using gas chromatography-olfactometry-mass spectrometry, odor activity values, and aroma recombination experiments. Foods 11 (14), 2106. 10.3390/foods11142106 35885354 PMC9319723

[B69] Li L. Zhang Y. Nan T. Yuan Y. (2025). GC-MS analysis of volatile organic compounds in Gastrodia elata during growth: characterization of odor-active compounds associated with horse urine odor. Food Biosci. 69, 106958. 10.1016/j.fbio.2025.106958

[B70] Lin C. J. Wu C. J. Pan C. F. Chen Y. C. Sun F. J. Chen H. H. (2010). Serum protein-bound uraemic toxins and clinical outcomes in haemodialysis patients. Nephrol. Dial. Transpl. 25 (11), 3693–3700. 10.1093/ndt/gfq251 20466687

[B71] Lin H. H. Huang C. C. Lin T. Y. Lin C. Y. (2015). p-Cresol mediates autophagic cell death in renal proximal tubular cells. Toxicol. Lett. 234 (1), 20–29. 10.1016/j.toxlet.2015.02.003 25668154

[B72] Liu W. C. Tomino Y. Lu K. C. (2018). Impacts of indoxyl sulfate and p-Cresol sulfate on chronic kidney disease and mitigating effects of AST-120. Toxins 10 (9), 367. 10.3390/toxins10090367 30208594 PMC6162782

[B73] Losch F. Weigend M. (2024). Diurnal patterns of floral volatile emissions in three species of narcissus. Am. J. Bot. 111 (10), e16408. 10.1002/ajb2.16408 39305022

[B74] Lu R. Fang Y. Wu W. Zeng X. Liu T. Qian Y. (2024). Hemodiafiltration with endogenous reinfusion for uremic toxin removal in patients undergoing maintenance hemodialysis: a pilot study. Ren. Fail 46 (1), 2338929. 10.1080/0886022X.2024.2338929 38632963 PMC11028005

[B75] Lugano R. Vemuri K. Barbera S. Orlandini M. Dejana E. Claesson-Welsh L. (2023). CD93 maintains endothelial barrier function by limiting the phosphorylation and turnover of VE-cadherin. FASEB J. 37 (4), e22894. 10.1096/fj.202201623RR 36961390 PMC11977521

[B76] Manikkaraja C. Bhavika M. Singh R. Nagarathnam B. George G. Gulyani A. (2022). Molecular and functional characterization of buffalo nasal epithelial odorant binding proteins and their structural insights by *in silico* and biochemical approaches. J. Biomol. Struct. Dyn. 40 (9), 4164–4187. 10.1080/07391102.2020.1854117 33292066

[B77] Mankhong S. Den-Udom T. Tanawattanasuntorn T. Suriyun T. Muta K. Kitiyakara C. (2024). The microbial metabolite p-cresol compromises the vascular barrier and induces endothelial cytotoxicity and inflammation in a 3D human vessel-on-a-chip. Sci. Rep. 14 (1), 18553. 10.1038/s41598-024-69124-w 39122790 PMC11316076

[B78] Mansurov A. Hosseinchi P. Chang K. Lauterbach A. L. Gray L. T. Alpar A. T. (2022). Masking the immunotoxicity of interleukin-12 by fusing it with a domain of its receptor *via* a tumour-protease-cleavable linker. Nat. Biomed. Eng. 6 (7), 819–829. 10.1038/s41551-022-00888-0 35534574 PMC11155269

[B79] Maremonti F. Meyer C. Linkermann A. (2022). Mechanisms and models of kidney tubular necrosis and nephron loss. ournal Am. Soc. Nephrol. 33 (3), 472–486. 10.1681/ASN.2021101293 35022311 PMC8975069

[B80] Marshall A. McGrath J. W. Graham R. McMullan G. (2023). Food for thought-the link between Clostridioides difficile metabolism and pathogenesis. PLoS Pathog. 19 (1), e1011034. 10.1371/journal.ppat.1011034 36602960 PMC9815643

[B81] Mayo B. Rodriguez J. Vazquez L. Florez A. B. (2021). Microbial interactions within the cheese ecosystem and their application to improve quality and safety. Foods 10 (3), 602. 10.3390/foods10030602 33809159 PMC8000492

[B82] Meier C. J. Nguyen M. N. Potter C. J. (2025). Making scents of mosquito repellents. Mak. scents mosquito repellents 41 (4), 280–289. 10.1016/j.pt.2025.02.009 40068978 PMC11968242

[B83] Meijers B. K. Claes K. Bammens B. de Loor H. Viaene L. Verbeke K. (2010). p-Cresol and cardiovascular risk in mild-to-moderate kidney disease. Clin. J. Am. Soc. Nephrol. 5 (7), 1182–1189. 10.2215/CJN.07971109 20430946 PMC2893077

[B84] Merkel C. D. Li Y. Raza Q. Stolz D. B. Kwiatkowski A. V. (2019). Vinculin anchors contractile actin to the cardiomyocyte adherens junction. Mol. Biol. Cell 30 (21), 2639–2650. 10.1091/mbc.E19-04-0216 31483697 PMC6761764

[B85] Meyer T. W. Peattie J. W. Miller J. D. Dinh D. C. Recht N. S. Walther J. L. (2007). Increasing the clearance of protein-bound solutes by addition of a sorbent to the dialysate. J. Am. Soc. Nephrol. 18 (3), 868–874. 10.1681/ASN.2006080863 17251385

[B86] Mlynarska E. Budny E. Saar M. Wojtanowska E. Jankowska J. Marciszuk S. (2024). Does the composition of gut microbiota affect chronic kidney disease? Molecular mechanisms contributed to decreasing glomerular filtration rate. Int. J. Mol. Sci. 25 (19), 10429. 10.3390/ijms251910429 39408756 PMC11477184

[B87] Moon S. J. Kim H. J. Cho S. B. Lee S. H. Choi H. Y. Park H. C. (2014). Epidermal proteinase-activated Receptor-2 expression is increased in end-stage renal disease patients with pruritus: a pilot study. Electrolyte and Blood Press. 12 (2), 74–79. 10.5049/EBP.2014.12.2.74 25606046 PMC4297706

[B88] Mozuraitis R. Buda V. Kutra J. Borg-Karlson A. K. (2012). p- and m-Cresols emitted from estrous urine are reliable volatile chemical markers of ovulation in mares. Animal Reproduction Sci. 130 (1-2), 51–56. 10.1016/j.anireprosci.2011.12.008 22266248

[B89] Mulatier M. Boullis A. Vega-Rua A. (2022). Semiochemical oviposition cues to control *Aedes aegypti* gravid females: state of the art and proposed framework for their validation. Parasit. Vectors 15 (1), 228. 10.1186/s13071-022-05337-0 35752845 PMC9233825

[B90] Muthukumar S. Muniasamy S. Srinivasan M. Ilangovan A. Satheshkumar S. Rajagopal T. (2018). Evaluation of pheromone-based kit: a noninvasive approach of estrus detection in buffalo. Reprod. Domest. Anim. 53 (6), 1466–1472. 10.1111/rda.13281 30043999

[B91] Nan W. He Y. Wang S. Zhang Y. (2023). Molecular mechanism of VE-cadherin in regulating endothelial cell behaviour during angiogenesis. Front. Physiol. 14, 1234104. 10.3389/fphys.2023.1234104 37601629 PMC10433914

[B92] National Center for Environmental Assessment (1988). 4- Methylphenol; CASRN 106-44-5. Chemical Assessment Summary. Integrated Risk Information System (IRIS). Washington, DC: United States Environmental Protection Agency. Available online at: https://cfpub.epa.gov/ncea/iris/iris_documents/documents/subst/0302_summary.pdf.

[B93] National Food Safety Standard: Standard for the Use of Food Additives (2024). (vol. GB2760-2024): National health commission of the People'S Republic of China and state administration for market regulation.

[B94] Nielsen M. S. Opbergen C. J. M. V. Veen T. A. B. V. Delmar M. (2023). The intercalated disc: a unique organelle for electromechanical synchrony in cardiomyocytes. Physiol. Rev. 103, 2271–2319. 10.1152/physrev.00021.2022 36731030 PMC10191137

[B95] Noh H. Yu M. R. Kim H. J. Jeon J. S. Kwon S. H. Jin S. Y. (2012). Uremia induces functional incompetence of bone marrow-derived stromal cells. Nephrol. Dial. Transpl. 27 (1), 218–225. 10.1093/ndt/gfr267 21622994

[B96] Noh H. Yu M. R. Kim H. J. Jang E. J. Hwang E. S. Jeon J. S. (2014). Uremic toxin p-cresol induces akt-pathway-selective insulin resistance in bone marrow-derived mesenchymal stem cells. Stem Cells 32 (9), 2443–2453. 10.1002/stem.1738 24805773

[B97] Norman B. P. Davison A. S. Hickton B. Ross G. A. Milan A. M. Hughes A. T. (2022). Comprehensive biotransformation analysis of phenylalanine-tyrosine metabolism reveals alternative routes of metabolite clearance in nitisinone-treated alkaptonuria. Metabolites 12 (10), 927. 10.3390/metabo12100927 36295829 PMC9611790

[B98] Oladi-Ghadikolaei R. Aliasgharzadeh A. Shayanfar A. Soleymani J. Moradi M. Jouyban A. (2023). Serum levels of indoxyl sulfate and P-cresol in type II diabetic patients with and without nephropathy. Iran. J. Kidney Dis. 17 (3), 126–134. 10.52547/ijkd.7266 37337796

[B99] Opdebeeck B. Maudsley S. Azmi A. De Mare A. De Leger W. Meijers B. (2019). Indoxyl sulfate and p-Cresyl sulfate promote vascular calcification and associate with glucose intolerance. J. Am. Soc. Nephrol. 30 (5), 751–766. 10.1681/ASN.2018060609 30940651 PMC6493976

[B100] Opdebeeck B. D'Haese P. C. Verhulst A. (2020). Molecular and cellular mechanisms that induce arterial calcification by indoxyl sulfate and P-Cresyl sulfate. Toxins 12 (1), 58. 10.3390/toxins12010058 31963891 PMC7020422

[B101] Osada K. Kashiwayanagi M. Izumi H. (2009). Profiles of volatiles in Male rat urine: the effect of puberty on the female attraction. Chem. Senses 34 (8), 713–721. 10.1093/chemse/bjp058 19759362

[B102] Oshiro S. Ramalho M. Duraes P. C. Andrade R. Silva K. Silverio F. (2020). p-Cresol in cheese: is it a flavouring compound or chemical contaminant? Food Addit. and Contam. Part A Chem. Analysis, Control, Expo. and Risk Assess. 37 (9), 1510–1519. 10.1080/19440049.2020.1778190 32679005

[B103] Pan L. Ye X. Ding J. Zhou Y. (2017). Antiproliferation effect of the uremic toxin para-cresol on endothelial progenitor cells is related to its antioxidant activity. Mol. Med. Rep. 15 (4), 2308–2312. 10.3892/mmr.2017.6230 28260040 PMC5364822

[B104] Pascucci T. Colamartino M. Fiori E. Sacco R. Coviello A. Ventura R. (2020). P-cresol alters brain dopamine metabolism and exacerbates autism-like behaviors in the BTBR mouse. Brain Sci. 10 (4), 233. 10.3390/brainsci10040233 32294927 PMC7226382

[B105] Passmore I. J. Letertre M. P. M. Preston M. D. Bianconi I. Harrison M. A. Nasher F. (2018). Para-cresol production by *Clostridium difficile* affects microbial diversity and membrane integrity of Gram-negative bacteria. PLoS Pathog. 14 (9), e1007191. 10.1371/journal.ppat.1007191 30208103 PMC6135563

[B106] Patel K. P. Luo F. J. Plummer N. S. Hostetter T. H. Meyer T. W. (2012). The production of p-cresol sulfate and indoxyl sulfate in vegetarians *versus* omnivores. Clin. J. Am. Soc. Nephrol. 7 (6), 982–988. 10.2215/CJN.12491211 22490877 PMC3362314

[B107] Peng Y. S. Ding H. C. Lin Y. T. Syu J. P. Chen Y. Wang S. M. (2012). Uremic toxin p-cresol induces disassembly of gap junctions of cardiomyocytes. Toxicology 302 (1), 11–17. 10.1016/j.tox.2012.07.004 22813906

[B108] Peng Y. S. Lin Y. T. Wang S. D. Hung K. Y. Chen Y. Wang S. M. (2013). P-cresol induces disruption of cardiomyocyte adherens junctions. Toxicology 306, 176–184. 10.1016/j.tox.2013.02.015 23466501

[B109] Persico A. M. Napolioni V. (2013). Urinary p-cresol in autism spectrum disorder. Neurotoxicol Teratol. 36, 82–90. 10.1016/j.ntt.2012.09.002 22975621

[B110] Poesen R. Evenepoel P. de Loor H. Kuypers D. Augustijns P. Meijers B. (2016). Metabolism, protein binding, and renal clearance of microbiota–derived p-Cresol in patients with CKD. Clin. J. Am. Soc. Nephrol. 11 (7), 1136–1144. 10.2215/cjn.00160116 27084876 PMC4934829

[B111] Rajanarayanan S. Archunan G. (2011). Identification of urinary sex pheromones in female buffaloes and their influence on bull reproductive behaviour. Res. Veterinary Sci. 91 (2), 301–305. 10.1016/j.rvsc.2010.12.005 21316068

[B112] Saito Y. Sato T. Nomoto K. Tsuji H. (2018). Identification of phenol- and p-cresol-producing intestinal bacteria by using media supplemented with tyrosine and its metabolites. FEMS Microbiol. Ecol. 94 (9), fiy125. 10.1093/femsec/fiy125 29982420 PMC6424909

[B113] Sato E. Hosomi K. Sekimoto A. Mishima E. Oe Y. Saigusa D. (2020). Effects of the oral adsorbent AST-120 on fecal p-cresol and indole levels and on the gut microbiota composition. Biochem. Biophys. Res. Commun. 525 (3), 773–779. 10.1016/j.bbrc.2020.02.141 32147096

[B114] Scholtes C. Nizet S. Collin S. (2014). Guaiacol and 4-methylphenol as specific markers of torrefied malts. Fate of volatile phenols in special beers through aging. J. Agric. Food Chem. 62 (39), 9522–9528. 10.1021/jf5015654 25174984

[B115] Shaw W. (2024). Hypothesis: 2 major environmental and pharmaceutical factors—acetaminophen exposure and gastrointestinal overgrowth of Clostridia bacteria induced by ingestion of glyphosate-contaminated foods—dysregulate the developmental protein sonic hedgehog and are major causes of autism. Integr. Med. 23 (3), 12–23. 39114279 PMC11302971

[B116] Shen Y. Wang Y. Shi Y. Bi X. Xu J. Zhu Q. (2020). Improving the clearance of protein-bound uremic toxins using cationic liposomes as an adsorbent in dialysate. Colloids Surf. B Biointerfaces 186, 110725. 10.1016/j.colsurfb.2019.110725 31862563

[B117] Shi Y. Zhang J. He J. Liu D. Meng X. Huang T. (2019). A method of detecting two tumor markers (p-hydroxybenzoic acid and p-cresol) in human urine using a porous magnetic <beta>-cyclodextrine polymer as solid phase extractant, an alternative for early gastric cancer diagnosis. Talanta 191, 133–140. 10.1016/j.talanta.2018.08.036 30262042

[B118] Singh R. K. Kumar S. Kumar S. Kumar A. (2008). Development of parthenium based activated carbon and its utilization for adsorptive removal of p-cresol from aqueous solution. J. Hazard. Mater. 155 (3), 523–535. 10.1016/j.jhazmat.2007.11.117 18207322

[B119] Soso S. B. Koziel J. A. (2017). Characterizing the scent and chemical composition of panthera leo marking fluid using solid-phase microextraction and multidimensional gas chromatography-mass spectrometry-olfactometry. Sci. Rep. 7 (1), 5137. 10.1038/s41598-017-04973-2 28698649 PMC5506057

[B120] Stachulski A. V. Knausenberger T. B. Shah S. N. Hoyles L. McArthur S. (2023). A host-gut microbial amino acid co-metabolite, p-cresol glucuronide, promotes blood-brain barrier integrity *in vivo* . Tissue Barriers 11 (1), 2073175. 10.1080/21688370.2022.2073175 35596559 PMC9870004

[B121] Suriyaphan O. Drake M. Chen X. Q. Cadwallader K. R. (2001). Characteristic aroma components of British farmhouse cheddar cheese. J. Agric. Food Chem. 49 (3), 1382–1387. 10.1021/jf001121l 11312868

[B122] Tang C. Cai J. Yin X. M. Weinberg J. M. Venkatachalam M. A. Dong Z. (2021). Mitochondrial quality control in kidney injury and repair. Nat. Rev. Nephrol. 17 (5), 299–318. 10.1038/s41581-020-00369-0 33235391 PMC8958893

[B123] Turriziani L. Ricciardello A. Cucinotta F. Bellomo F. Turturo G. Boncoddo M. (2022). Gut mobilization improves behavioral symptoms and modulates urinary p-cresol in chronically constipated autistic children: a prospective study. Autism Res. 15 (1), 56–69. 10.1002/aur.2639 34813183 PMC9299106

[B124] Ueno T. Kiyohara S. Ho C.-T. Masuda H. (2006). Potent inhibitory effects of black tea theaflavins on off-odor formation from citral. J. Agric. Food Chem. 54 (8), 3055–3061. 10.1021/jf052803h 16608230

[B125] Ujhelyi L. Balla G. Jeney V. Varga Z. Nagy E. Vercellotti G. M. (2006a). Hemodialysis reduces inhibitory effect of plasma ultrafiltrate on LDL oxidation and subsequent endothelial reactions. Kidney Int. 69 (1), 144–151. 10.1038/sj.ki.5000007 16374435

[B126] Ujhelyi L. Balla G. Jeney V. Varga Z. Nagy E. Vercellotti G. M. (2006b). Response to p-Cresol for better or worse: but what are we measuring? Kidney Int. 70 (1), 232–233. 10.1038/sj.ki.5001531 16810293

[B127] Unno T. Sakuma M. Mitsuhashi S. (2014). Effect of dietary supplementation of (-)-epigallocatechin gallate on gut microbiota and biomarkers of colonic fermentation in rats. J. Nutr. Sci. Vitaminology 60 (3), 213–219. 10.3177/jnsv.60.213 25078378

[B128] Vacca M. Celano G. Lenucci M. S. Fontana S. Forgia F. M. Minervini F. (2021). *In vitro* selection of probiotics, prebiotics, and antioxidants to develop an innovative synbiotic (NatuREN G) and testing its effect in reducing uremic toxins in fecal batches from CKD patients. Microorganisms 9 (6), 1316. 10.3390/microorganisms9061316 34204263 PMC8235484

[B129] Valim A. Carpes L. S. Nicoletto B. B. (2022). Effect of vegetarian diets on renal function in patients with chronic kidney disease under non-dialysis treatment: a scoping review. J. Bras. Nefrol. 44 (3), 395–402. 10.1590/2175-8239-JBN-2021-0126 35138323 PMC9518626

[B130] Vanholder R. Baurmeister U. Brunet P. Cohen G. Glorieux G. Jankowski J. (2008). A bench to bedside view of uremic toxins. J. Am. Soc. Nephrol. 19 (5), 863–870. 10.1681/ASN.2007121377 18287557

[B131] Varinder S. Rupinder K. Pratima K. Chirag P. Ravinder S. (2023). ICAM-1 and VCAM-1: gatekeepers in various inflammatory and cardiovascular disorders. Clin. chimica acta; Int. J. Clin. Chem. 548, 117487. 10.1016/j.cca.2023.117487 37442359

[B132] Velasquez M. T. Centron P. Barrows I. Dwivedi R. Raj D. S. (2018). Gut microbiota and cardiovascular uremic toxicities. Toxins 10 (7), 287. 10.3390/toxins10070287 29997362 PMC6071268

[B133] Versele R. Sevin E. Gosselet F. Fenart L. Candela P. (2022). TNF-α and IL-1β modulate blood-brain barrier permeability and decrease Amyloid-β peptide efflux in a human blood-brain barrier model. Int. J. Mol. Sci. 23 (18), 10235. 10.3390/ijms231810235 36142143 PMC9499506

[B134] Vijayasarathy M. Kiran G. K. Balaji S. Jabastin J. Bruntha Devi P. Brindha Priyadarisini V. (2020). *In vitro* detoxification studies of p-Cresol by intestinal bacteria isolated from human feces. Curr. Microbiol. 77 (10), 3000–3012. 10.1007/s00284-020-02124-x 32683469

[B135] Wang H. Ruan G. Li Y. Liu X. (2025). The role and potential application of IL-12 in the immune regulation of tuberculosis. Int. J. Mol. Sci. 26 (7), 3106. 10.3390/ijms26073106 40243848 PMC11988481

[B136] Weir M. R. Bakris G. L. Bushinsky D. A. Mayo M. R. Garza D. Stasiv Y. (2015). Patiromer in patients with kidney disease and hyperkalemia receiving RAAS inhibitors. N. Engl. J. Med. 372 (3), 211–221. 10.1056/NEJMoa1410853 25415805

[B137] Wong X. Carrasco-Pozo C. Escobar E. Navarrete P. Blachier F. Andriamihaja M. (2016). Deleterious effect of p-Cresol on human colonic epithelial cells prevented by proanthocyanidin-containing polyphenol extracts from fruits and proanthocyanidin bacterial metabolites. J. Agric. Food Chem. 64 (18), 3574–3583. 10.1021/acs.jafc.6b00656 27039931

[B138] Xia Y. Hu L. Ren K. Han X. Sun Y. Li D. (2025). Embryonic exposure to 6:2 fluorotelomer alcohol mediates autism spectrum disorder-like behavior by dysfunctional microbe-gut-brain axis in mice. J. Hazard. Mater. 484, 136739. 10.1016/j.jhazmat.2024.136739 39637794

[B139] Yamakoshi J. Tokutake S. Kikuchi M. Kubota Y. Konishi H. Mitsuoka T. (2009). Effect of proanthocyanidin-rich extract from grape seeds on human fecal flora and fecal odor. Microb. Ecol. Health Dis. 13 (1), 25–31. 10.1080/089106001750071672

[B140] Yan Z. Zhong H. M. Maher N. Torres R. Leo G. C. Caldwell G. W. (2005). Bioactivation of 4-methylphenol (p-cresol) *via* cytochrome P450-mediated aromatic oxidation in human liver microsomes. Drug Metab. Dispos. 33 (12), 1867–1876. 10.1124/dmd.105.006387 16174805

[B141] Yeung S. Y. Lan W. H. Huang C. S. Lin C. P. Chana C. P. Chang M. C. (2002). Scavenging property of three cresol isomers against H2O2, hypochlorite, superoxide and hydroxyl radicals. Food Chem. Toxicol. 40 (10), 1403–1413. 10.1016/s0278-6915(02)00102-3 12387302

[B142] Yoon Y. M. Han Y. S. Yun C. W. Lee J. H. Kim R. Lee S. H. (2018). Pioglitazone protects mesenchymal stem cells against P-Cresol-Induced mitochondrial dysfunction *via* Up-Regulation of PINK-1. Int. J. Mol. Sci. 19 (10), 2898. 10.3390/ijms19102898 30250007 PMC6213327

[B143] Zare F. Janeca A. Jokar S. M. Faria M. Goncalves M. C. (2022). Interaction of human serum albumin with uremic toxins: the need of new strategies aiming at uremic toxins removal. Membr. (Basel) 12 (3), 261. 10.3390/membranes12030261 35323736 PMC8953794

[B144] Zayed M. Iohara K. (2020). Effects of p-Cresol on senescence, survival, inflammation, and odontoblast differentiation in canine dental pulp stem cells. Int. J. Mol. Sci. 21 (18), 6931. 10.3390/ijms21186931 32967298 PMC7555360

[B145] Zhao J. Munanairi A. Liu X. Y. Zhang J. Hu L. Hu M. (2020). PAR2 mediates itch *via* TRPV3 signaling in keratinocytes. J. Invest Dermatol 140 (8), 1524–1532. 10.1016/j.jid.2020.01.012 32004565 PMC7387154

[B146] Zheng Y. Prince N. Z. Peralta Marzal L. N. Ahmed S. Garssen J. Perez Pardo P. (2022). The autism spectrum disorder-associated bacterial metabolite p-Cresol derails the neuroimmune response of microglial cells partially *via* reduction of ADAM17 and ADAM10. Int. J. Mol. Sci. 23 (19), 11013. 10.3390/ijms231911013 36232346 PMC9570133

[B147] Zhu J. Z. Zhang J. Yang K. Du R. Jing Y. J. Lu L. (2012). P-cresol, but not p-cresylsulphate, disrupts endothelial progenitor cell function *in vitro* . Nephrol. Dial. Transpl. 27 (12), 4323–4330. 10.1093/ndt/gfs382 22962408

[B148] Zhu F. Xu P. Barbosa R. M. Choo Y. M. Leal W. S. (2013). RNAi-based demonstration of direct link between specific odorant receptors and mosquito oviposition behavior. Insect Biochem. Mol. Biol. 43 (10), 916–923. 10.1016/j.ibmb.2013.07.008 23911547 PMC3800558

[B149] Zhu N. Duan H. Feng Y. Xu W. Shen J. Wang K. (2023). Magnesium lithospermate B ameliorates diabetic nephropathy by suppressing the uremic toxin formation mediated by gut microbiota. Eur. J. Pharmacol. 953, 175812. 10.1016/j.ejphar.2023.175812 37245856

[B150] Zhuang H. Liang Z. Ma G. Qureshi A. Ran X. Feng C. (2024). Autism spectrum disorder: pathogenesis, biomarker, and intervention therapy. Med. Comm (2020) 5 (3), e497. 10.1002/mco2.497 38434761 PMC10908366

